# Machine learning applications for predicting safety incidents in construction industry

**DOI:** 10.1038/s41598-025-34763-0

**Published:** 2026-01-10

**Authors:** Saleh Alsulamy, Mohamed Alshayeb, Inamullah Inam, Anwar Ahmed

**Affiliations:** 1 Architecture Department, College of Architecture and Planning Department, King Khalid University, Asser, 394, Abha, Kingdom of Saudi Arabia; 2 Department of Civil Engineering, Engineering Faculty, Laghman University, Mehtarlam, Afghanistan; 3 Department of Civil Engineering, College of Engineering, Northern Border University, Arar, 73222 Saudi Arabia

**Keywords:** Safety incidents, Construction industry, Fatality rate, Machine learning, Engineering, Civil engineering

## Abstract

Construction site accidents pose serious risks to workers and organizations, necessitating proactive measures to mitigate fatalities and severe injuries. Identifying key contributing factors and developing predictive models are essential. Whereas earlier studies relied on questionnaires or basic statistics and often focused on a single outcome, this study develops machine learning (ML) models that jointly address both the nature of incidents (NOI) and the severity of incidents (SOI). A dataset of 203 incidents from Saudi Arabia (2018–2024), comprising 14 explanatory variables, was used. Six ML algorithms, K-Nearest Neighbor (KNN), Support Vector Machine (SVM), Decision Tree (DT), Random Forest (RF), Gradient Boosting (GB), and Extreme Gradient Boosting (XGB) were employed. Results show that all models achieved over 60% accuracy, with XGB performing best, reaching 89% accuracy in SOI prediction. Incorporating NOI as an explanatory feature further improved SOI prediction, highlighting the interdependence of incident characteristics and severity. SHAP analysis provided interpretable insights, revealing that NOI, precipitation, date of incident, and workforce size were the most influential predictors. Factors such as location, safety training, and PPE compliance contributed additional explanatory power. These findings provide practical insights for construction firms, supporting the development of targeted emergency response strategies and the efficient deployment of first-aid resources on site.

## Introduction

 The construction sector consistently intersects with health and safety concerns, given its substantial workforce. Fatal injuries are prevalent in this industry, with construction workers experiencing a higher risk of both fatal and non-fatal injuries compared to other occupational groups. Although countries differ in industrial structure and occupational health and safety standards, construction-related injuries are a primary concern in many nations. In 2019, the construction sector in the United States experienced over 1000 worker fatalities, accounting for nearly 20% of all worker fatalities in the private business^1^. Similarly, the UK construction industry experiences a statistically higher incidence of fatal injuries, with the fatality rate for construction workers being four times that of the overall industry average, as stated by the Health and Safety Executive in 2023^2^. Another example is of Iran. As a developing nation, Iran boasts numerous construction firms. Statistical data reveals that construction site accidents in the country have led to significant human and socioeconomic losses^3^. Despite construction employing less than 12% of Iranian workers, the severity of injuries remains remarkably high^4^.

To prevent the repetition of accidents, it is imperative to carry out post-investigations, which facilitate the integration of acquired knowledge into plans aimed at mitigating their impact^5^. Hence, conducting a comprehensive and evaluative examination of incidents occurring within the construction sector carries substantial importance.

Recently, there has been a significant transformation in the construction industry, characterized using Artificial Intelligence (AI) and Machine Learning (ML) approaches. In the construction business, the implementation of these technologies has resulted in a substantial revolution in decision-making procedures^6,7^. Advanced decision support systems (DSS) equipped with AI capabilities are made available to building experts^8^. In this context, DSS provide decision-makers with the ability to improve project results, increase efficiency, and reduce costs. They do so by automating cyclic processes, improving resource allocation, and identifying potential dangers^9^. Construction engineering greatly benefits from the use of AI and ML approaches. These methods can effectively analyse intricate information, identify valuable patterns, and produce insightful findings. The utilization of human expertise and intuition in traditional decision-making procedures renders them vulnerable to biases and limits^9^. On the other hand, ML algorithms effectively analyse vast quantities of organized and unstructured data, revealing complicated correlations and delivering precise forecasts^10^. These technological advancements enhance the cognitive capacities of experts in the construction field and help them use data more effectively for strategic choices^11^.

Furthermore, ML models are becoming increasingly significant in the construction business. By embracing AI and ML, construction can achieve significant improvements in managing costs, schedules, and worker safety. For instance, Kannan et. al^12^study on using ML to predict production delays in quarries. Analysis of 6 months of data from 2 machines identified Neural Networks and Logistic Regression as most accurate for predicting delays (F-measure of 0.973). This can improve decision-making and reduce production stoppages. Furthermore, Samy et. al^13^. explored using ML to forecast hospital period of staying for improved resource management in construction companies. Analysing MIMIC III data, a random forest (RF) model achieved 90% accuracy in predicting LOS, demonstrating the potential of ML for optimizing resource allocation and health safety policies in construction.

Traditional post-investigation methods rely on predefined statistical models and expert interpretations and mainly provide descriptive insights into past incidents. In contrast, ML approaches can analyse large, complex datasets to uncover hidden patterns and predict incident patterns with higher accuracy by identifying key contributing factors such as PPE usage, precipitation, and the nature of the incident. Additionally, ML enhances post-investigations by automating the detection of emerging risk trends, improving the reliability of safety assessments, and allowing for proactive risk mitigation strategies. These advancements position ML as a powerful complement to traditional construction safety practices.

### Related work and research significance

Recent work has begun to apply ML to construction safety, moving beyond conventional post-hoc or purely descriptive analyses toward predictive modelling of incident outcomes^14,15^. Unlike traditional approaches that rely on predefined models and expert judgment, ML can process large, heterogeneous datasets and uncover non-obvious patterns related to site conditions, human factors, and context. These capabilities support more proactive risk mitigation in practice^14,15^.

A first stream of studies targets fatality risk. Choi et al.^16^ used Logistic Regression (LR), Decision Trees (DT), and RF to estimate the probability of fatal outcomes from personnel- and project-level descriptors (e.g., age, tenure, construction type, workforce size, temporal factors). While influential, this study omitted injury-level variables, a limitation that may constrain predictive fidelity^16^. Building on the fatality focus, Mohammed and Mahmud^17^ classified incidents as fatal vs. non-fatal using gradient boosting trees, RF, and DT. They reported encouraging accuracy but offered limited analysis of which factors most strongly drive fatal outcomes. Complementing these efforts, Assaad and El-Adaway^18^ applied spectral clustering on incident reports to reveal salient contributors to mortality. However, generalizability was restricted by a small dataset, and the work did not develop predictive models.

A second stream emphasizes injury and incident categorization from large text corpora. Baker et al.^19^ leveraged natural-language features and model ensembles (e.g., RF, XGB, linear SVM, stacking) for injury classification over a large repository of narrative reports. Their results highlighted the value of richer feature extraction for prediction. Relatedly, Kang and Ryu^20^ modelled accident-type classification with RF and identified salient predictors (e.g., improper PPE, unclean workspace), underscoring the operational relevance of site practices for risk reduction.

Broader occupational safety and health research still frequently relies on survey-driven descriptive statistics or basic inferential models to explain injury severity and its correlates. This body of work illustrates both the strengths and limitations of non-ML approaches (e.g., large-scale analyses using cross-tabulations and logistic regression)^21^. Together, these strands show clear momentum toward data-driven safety analytics while also revealing methodological and scope gaps. Table 1 provides a concise synthesis of recent ML-based studies on construction-incident prediction.

**Table 1:** Summary of recent machine learning studies on construction-safety incident prediction.

Study	Data and context	Target(s)	Methods	Key Variables/Insights	Performance Metric(s)
Choi et al^16^.	National records; 137,323 injuries & 2,846 fatalities	Fatality risk (binary)	LR, DT, RF, AdaBoost	RF performed best in classifying high-risk fatality cases. Season, employer size, and age were major predictors.	91.98% (AUC for RF)
Mohammed & Mahmud^17^	Construction incident records	Fatal vs. non-fatal classification	GB, RF, DT	Exploratory focus on predictive accuracy; limited analysis of drivers of fatality risk	Accuracy ≈ 70%
Assaad & El-Adaway^18^	100 incident reports	Clustering related to mortality contributors (unsupervised)	Spectral clustering	Identified contributors to mortality; generalizability limited by small dataset; no predictive model	-
Baker et al^19^.	90,000 + incident reports	Injury/incident classification	RF, XGB, Linear SVM, Model Stacking	Richer NLP features improved prediction; value of large text corpora for safety analytics	Accuracy ≈ 42–65%
Kang and Ryu^20^	6,374 incidents	Accident type classification (e.g., falls, collisions)	RF	Improper PPE and unclean workspace were major contributors	Accuracy ≈ 71.3%

Based on the above research gaps, there is a clear need for approaches that can simultaneously address multiple construction safety outcomes while ensuring interpretability for practical decision-making. Most prior studies have examined a single outcome such as fatality risk, accident type, or severity without linking them or explaining feature contributions in a transparent way. To address these shortcomings, this study has the following objectives:

- Develop predictive models for both the nature of incident (NOI) and the severity of incident (SOI).
- Incorporate NOI as an explanatory feature to evaluate its impact on improving SOI prediction.
- Apply SHAP analysis to provide explainable insights into the most influential predictors of incident outcomes.
- Benchmark different machine learning algorithms within a unified evaluation process to compare their relative strengths for safety prediction tasks.
- Provide context-specific insights relevant to the Saudi Arabian construction industry, contributing to data-driven safety management practices.

To guide the reader, this manuscript is structured as follows: Sect. “Research methodology” describes the methodology, including data collection, preprocessing, multicollinearity assessments, and the machine learning algorithms employed. Section “Results and discussion” presents the results and discussion, covering model validation, performance evaluation, feature importance analysis, and SHAP-based interpretability. Section “Practical Implications, future research directions and research limitations” outlines practical implications, future research directions, and research limitations. Section “Conclusions” provides concluding remarks.

## Research methodology

### Data collection

The research study outlined in this paper comprised two primary segments. The initial phase focused on constructing a database of construction accidents for statistical analysis. This involved the collection of reports from an initial dataset of 250 construction accidents resulting in either fatal or nonfatal injuries. However, 47 incidents were excluded due to missing or incomplete data in critical explanatory variables, such as time with employer, time of incident, and specific PPE types. These exclusions were necessary to ensure data quality and reliability, leaving 203 incidents as the final dataset used for model development and analysis. The second phase of the study involved developing comprehensive ML models to predict the nature (NOI) and severity (SOI) of construction incidents, providing a useful framework for preventing future accidents.

For data collection, a structured questionnaire was developed and circulated (attached as Annex A). The dataset consists of construction-based safety incidents from Saudi Arabia, primarily from Makkah and Riyadh regions, covering incidents from 2018 to 2024.

The study categorizes safety incidents according to the Occupational Injury and Illness Classification System (OIICS)^22^.

1. Nature of incident: The statement delineates the primary physiological attributes of the injury. Several prevalent types of fatalities in the construction industry encompass bruises, falls, burns, fractures, electrical shock, and traffic accidents.
2. Severity level of incident: Numerical value representing the severity of the incident on a pre-defined scale of 1–5, where 1 is minor and 5 is fatal.

The OIICS^22^ offers comprehensive information regarding deaths, encompassing the physical attributes of the victims (such as the kind of the injury and the specific part of the body affected) as well as the origins and occurrences that lead to fatalities (including the cause of the injury and the incident or exposure). It is widely held that employing such a method, as opposed to relying just on a single dependent variable such as the risk of fatality, can yield more valuable insights and enhance the preparedness of pertinent stakeholders in comprehending and mitigating fatalities. Following a thorough analysis, data regarding each incident is gathered from the employer, coworkers present at the site, safety personnel, members of the onsite emergency response team, and other individuals who have witnessed the incident^22^. To minimize potential biases from subjective reporting, the collected data was cross validated using official injury logs maintained by site safety officers, ensuring consistency and accuracy in the reported details. Additionally, a standardized questionnaire was used during data collection to ensure uniformity in responses and reduce variability in subjective accounts. In cases of discrepancies between witness accounts and injury logs, priority was given to documented records to enhance data reliability. This study utilizes a set of input and output variables, which are presented in Table 2 with related numerical digit.

**Table 2:** Explanatory and target variables.

Input Variables	
Notation	Values
**X1**: Project Type	(1) Residential, (2) Commercial, (3) Industrial
**X2**: Project Size	(1) <$1 million, (2) $1–5 million, (3) $5–10 million, (4) >$10 million
**X3**: Project duration	(1) < 6 months, (2) 6–12 months, (3) 1–2 years, (4) >4 years
**X4**: Number of workers	Numerical value
**X5**: Subcontractor involvement	(1) Yes, (2) No
**X6**: Safety Training provided	(1) Yes, (2) No.
**X7**: PPE used	(1) Yes, (2) No.
**X8**: Temperature	(1) 0 °C, (2) 0 °C to 10 °C, (3) 10 °C to 20 °C, (4) 20 °C to 30 °C, (5) above 30 °C
**X9**: Wind speed	(1) Calm (less than 5 km/h), (2) Light breeze (5–15 km/h), (3) Moderate breeze (16–30 km/h), (4) Fresh breeze (31–49 km/h), (5) Strong breeze (50–61 km/h), (6) High wind (above 61 km/h)
**X10**: Precipitation	(1) None, (2) Light rain, (3) Moderate rain, (4) Heavy rain
**X11**: Worker age	(1) 18–25, (2) 26–35, (3) 36–45, (4) 45+
**X12**: Worker Experience	(1) 0–2 years, (2) 3–5 years, (3) 5–10 years, (4) 10 + years
**X13**: Incident Year	Numerical value
**X14**: Location of incident	(1) Scaffolding area, (2) Roof, (3) Excavation pit, (4) Electrical room, (5) Material storage room, (6) Workshop, (7) Road

Output Variables	
Notations	Values
NOI	Falls: 1 Cut/Bruise/Fracture/Foreign object in eye: 2. Traffic accident: 3. Crushed under moving object/under load: 4. Struck by falling object: 5. Fire, explosion: 6. Electrical shock: 7. Construction collapse: 8
SOI	Numerical value representing the severity of the incident on a pre-defined scale of 1–5, where 1 is minor and 5 is fatal

### Data preprocessing

Data preparation involved the following steps. First, the categorical output variables (NOI and SOI) were compiled and restructured according to the questionnaire. Following an extensive examination of existing literature^16,19,23^, a total of 17 explanatory factors were initially selected for predicting fatality severity. However, three variables, time with employer, fall height, and day/nighttime were excluded due to excessive missing data. The omission of fall height and day/nighttime is particularly noteworthy, as these factors could significantly influence incident severity. Fall height is directly correlated with injury severity in fall-related incidents, while day/nighttime conditions may impact visibility and worker fatigue, potentially increasing accident risks. Given their potential predictive value, imputation methods were explored to retain these variables. However, due to the high proportion of missing values, simple mean imputation risked introducing bias, and regression-based imputation did not yield reliable patterns due to data sparsity. Thus, these variables were excluded to maintain dataset integrity and avoid introducing artificial correlations. Future studies with more complete datasets could explore their predictive potential further. The remaining 14 variables have been retained for prediction. Additionally, NOI was incorporated as an explanatory feature to improve SOI prediction. Ultimately, any fatality data that contains missing figures is eliminated. Consequently, a comprehensive dataset of 203 fatalities has been established, encompassing 2 dependent variables and 14 explanatory variables.

#### Dataset overview and characteristics

**Fig. 1:**
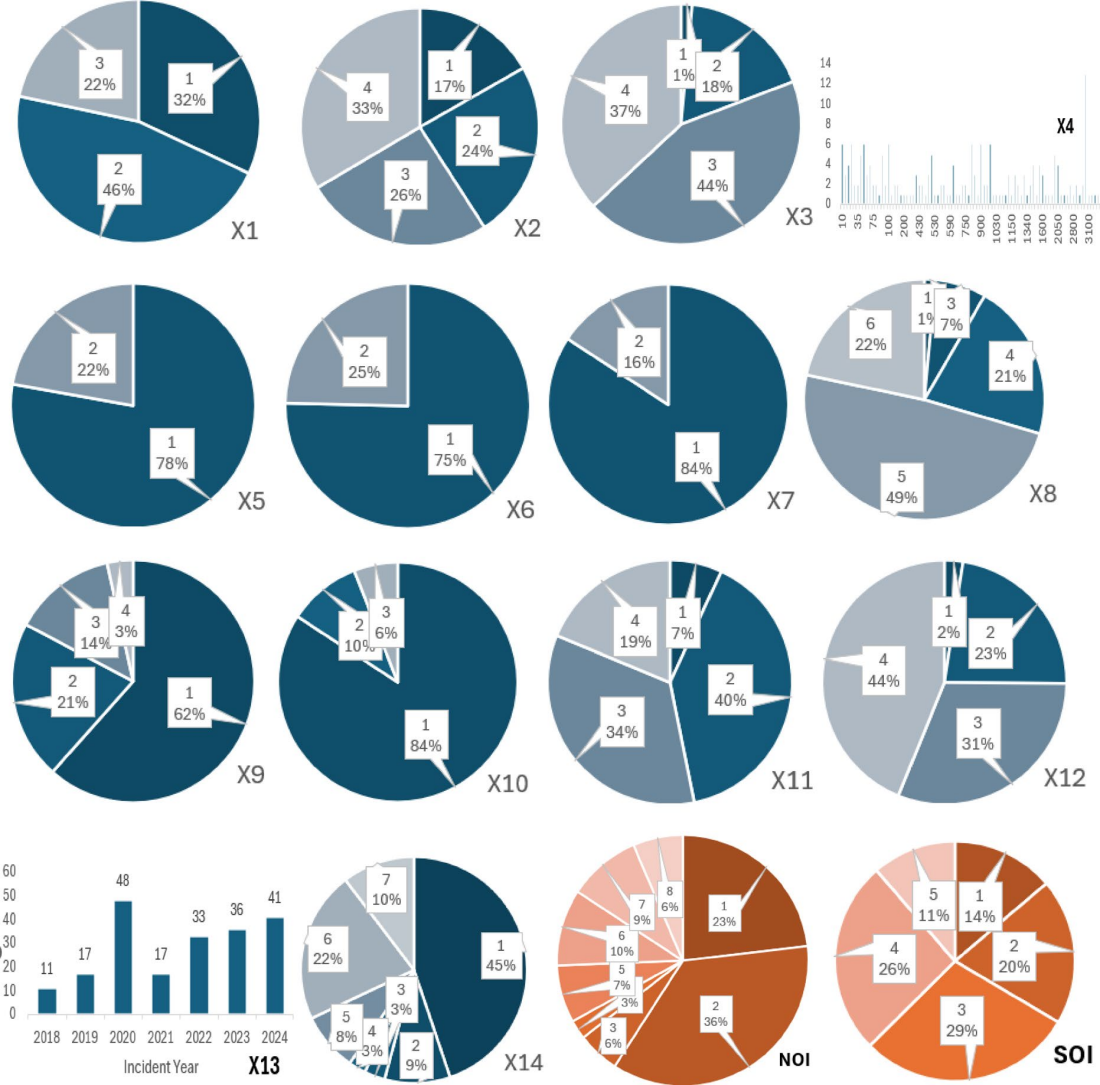
Quantitative attributes of the input and output variables.

To provide a clear understanding of the modelling dataset after encoding (Sect. “Encoding categorical data”), Table 3 presents the descriptive statistics of the 14 explanatory variables and the target (SOI). The table summarizes the mean, standard deviation, minimum, and maximum values, which help readers understand the distribution and scale of the encoded variables used in model training. These statistics establish the quantitative profile of the dataset and complement the categorical distributions illustrated in Fig. 1.

**Table 3:** Post-encoding descriptive statistics.

Features	Count	Mean	Standard Deviation	Minimum	Maximum
Project Type	203	1.8966	0.7272	1	3
Project Budget	203	2.7586	1.0927	1	4
Project duration	203	3.1626	0.7631	1	4
Number of Workers on Site	203	964.32	961.28	10	3350
Subcontractor Involvement	203	1.2217	0.4164	1	2
Relevant Safety Training	203	1.2463	0.4319	1	2
Appropriate PPE Used	203	1.1576	0.3653	1	2
Temperature	203	4.8079	0.9531	1	6
Wind Speed	203	1.5911	0.8533	1	4
Precipitation	203	1.2167	0.538	1	3
Age Range	203	2.6502	0.8622	1	4
Years of Experience	203	3.1626	0.8607	1	4
Location of Incident	203	3.266	2.4104	1	7
Nature of Incident	203	3.3399	2.3458	1	8
Severity of Incident	203	3.0148	1.2125	1	5

This analysis examines construction incidents reported between 2018 and 2024. Among the 203 incidents, the proportional distribution of severity levels, as defined by the SOI classification framework, is as follows:

- SOI Level 1 (Minor Incidents): 14%.
- SOI Level 2 (Moderate Incidents): 20%.
- SOI Level 3 (Severe but Non-Fatal Incidents): 29%.
- SOI Level 4 (Critical Incidents, Life-Threatening): 26%.
- SOI Level 5 (Fatal Incidents): 11%.

The distribution (Fig. 1) highlights that most incidents fall into moderate to severe categories (SOI Levels 2–4), while fatal incidents (SOI Level 5) represent a smaller yet critical portion of the dataset (11%). The most frequent injury type was mild, involving cuts, bruises, or fractures. Falls were the leading cause of these minor injuries, with scaffolding areas accounting for a significant portion (45%) as indicated by variable X14. Workshops were the second most common location for incidents (22% - X14), often involving multiple mishaps. Road areas followed at 10%, where traffic accidents were the primary cause of fatalities.

Examination of explanatory variables reveals concerning trends. A significant number of employers (25% - X6) failed to provide adequate safety training programs, and 16% (X7) neglected to enforce Personal Protective Equipment (PPE) use during construction. These gaps in employer practices, such as insufficient safety training and lack of PPE enforcement, may contribute to construction fatalities, as indicated by the reported deficiencies in these areas.

#### Handling imbalanced classes

The dataset exhibited a significant class imbalance, with fatalities (SOI Level 5) accounting for only 11% of the total incidents. Such imbalance can bias ML models toward majority classes, reducing their ability to predict minority outcomes like fatalities. To address this challenge, the Synthetic Minority Oversampling Technique (SMOTE)^24^ was applied during preprocessing to generate synthetic samples for the minority class (SOI Level 5). This technique works by creating new data points in the feature space based on existing samples, ensuring that the minority class is sufficiently represented during model training. Moreover, for models such as XGB and GB, class weights were assigned inversely proportional to class frequencies. This adjustment penalized misclassifications of the minority class, ensuring that fatalities were prioritized without overfitting.

#### Dataset partitioning and feature scaling

The dataset utilized for the development of different models was divided into two distinct groups: a training set, employed for training the algorithms, and a testing set, utilized for evaluating the performance of the algorithms. The dataset was partitioned using an 80 − 20 training-testing split, following methodologies established in previous literature. Furthermore, to ensure proper feature scaling, we applied standardization (mean = 0, standard deviation = 1) selectively to models that are sensitive to feature scales, such as SVM and KNN. However, tree-based models, including RF, DT, and XGB, are inherently scale-invariant and do not require standardization. For these models, raw feature values were used without transformation to avoid unnecessary computations and potential biases. This procedure facilitates the standardization of all features, hence enhancing convergence throughout the training phase. In addition, one hot encoding technique is utilized to transform category information into numerical representations^25–27^.

#### Encoding categorical data

To ensure compatibility with ML models, all categorical variables in the dataset, including NOI, PPE usage, incident location, weather conditions, and other explanatory factors, were transformed using Label Encoding. This method assigns a unique numerical value to each categorical class, allowing ML models to process the data efficiently without increasing dataset dimensionality.

Label Encoding was applied uniformly across both multi-class and binary categorical variables. For instance, in the case of NOI, each type of incident was assigned a numeric value (e.g., “Fall” = 0, “Collision” = 1, “Fire” = 2), ensuring that the variable could be effectively used in predictive modelling. Similarly, binary categorical features such as PPE usage (Yes/No) were also label-encoded (e.g., “No” = 0, “Yes” = 1), rather than employing one-hot encoding, which would have increased dataset complexity by creating multiple additional columns.

This approach was chosen because ML models like XGB, RF and DT inherently handle encoded numerical data, meaning that categorical features do not need additional transformation. By applying Label Encoding uniformly to all categorical data, the dataset was structured in a way that optimized both computational efficiency and predictive performance, ensuring that the models could effectively capture relationships between categorical variables and SOI.

#### Multicollinearity analysis

**Fig. 2:**
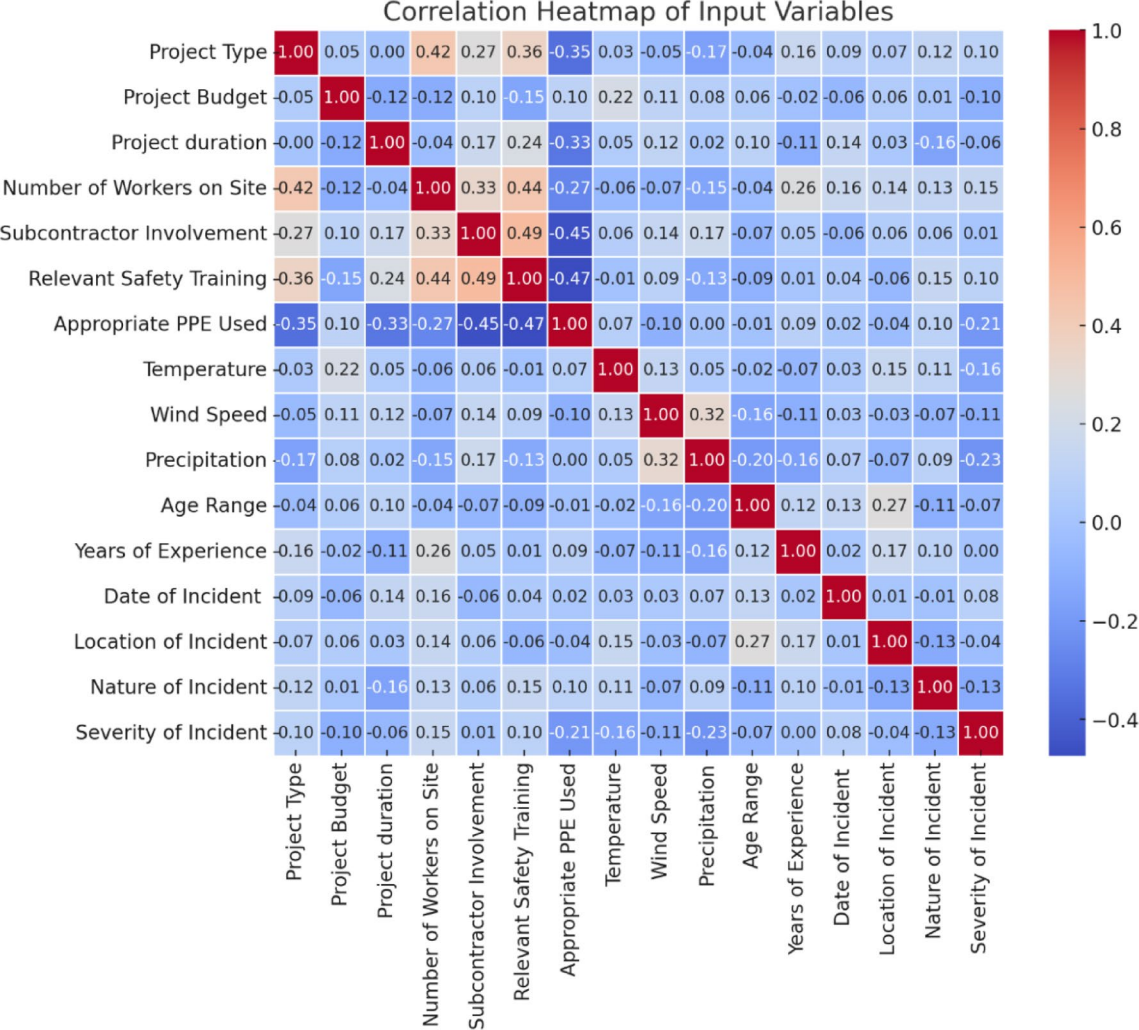
Correlation heatmap of input variables.

The importance of NOI in predicting SOI raises concerns about potential multicollinearity between NOI and other explanatory variables, therefore, a correlation analysis was conducted to assess relationships between explanatory variables. Figure 2 presents a correlation heatmap, illustrating the strength of associations among key features. The results indicate moderate correlations between NOI and PPE use, location, and precipitation, suggesting that while some dependencies exist, they are not strong enough to cause multicollinearity issues. The acceptable correlation threshold for avoiding multicollinearity concerns is typically below 0.70–0.80^28^. In this study, all feature correlations remained below 0.85, ensuring that the model’s feature importance analysis remains valid and interpretable. Since ML models like XGB and RF are inherently robust to correlated features, multicollinearity does not significantly affect their predictions. Unlike regression models, where high multicollinearity (correlation > 0.85) can distort coefficient estimates, tree-based models can handle feature dependencies through internal feature selection and importance weighting. Therefore, no further adjustments (such as feature removal or dimensionality reduction) were required. These results confirm the independent contributions of key variables like NOI, precipitation, and PPE use in predicting SOI while ensuring that multicollinearity remains within acceptable limits.

### Machine learning algorithms

For predicting such parameters, several ML models are employed to predict and classify such as K-Nearest Neighbours (KNN), Support Vector Machine (SVM), DT, RF, Gradient Boosting (GB) and Extreme Gradient Boosting (XGB). These models are conducted using the python language in Google Colab. The ML models, in conjunction with computational packages, have undergone significant advancements and have been successfully utilized in forecasting the safety incident features in the construction industry, as highlighted in prior studies^19,23,29–32^.

The rationale for selecting these models lies in their proven strengths as described in Table 1. XGB and RF were chosen for their ability to handle non-linear relationships and feature interactions, which are critical in analysing the complex factors contributing to safety incidents. DT was included for its simplicity and interpretability, while SVM and KNN are well-suited for smaller datasets, aligning with the size of this study’s dataset. Deep learning models, such as artificial neural networks (ANN), were not employed due to their high risk of overfitting when applied to small datasets^33^. The following sub sections describes about the models utilized in this study.

#### K nearest neighbour

In regression tasks, a non-parametric method called KNN regression can be useful. This approach forecasts the target value for a new data point by taking a mean of the output values of its K most similar neighbours within the training data. To determine how similar data points are, a distance metric, often Euclidean distance, is used^34^. The effectiveness of KNN hinges on selecting the appropriate values for K and the distance measure. The KNN algorithm offers several benefits when dealing with non-linear or complex data, as it does not rely on any assumptions on the specific functional relationship between characteristics and the target variable^35^.

For this study, the value of k (number of neighbours) was determined empirically by testing a small range of candidate values (3–9). The configuration with k = 5 consistently produced the most stable performance across evaluation metrics, balancing local sensitivity with generalization^36^. While this setting is effective for exploratory prediction, the model’s performance remains sensitive to the choice of k and the distance metric. The high dimensionality and class imbalance in the dataset posed additional challenges for KNN, which relies on local neighbourhoods for classification.

#### Support vector machine

SVMs are a prominent ML technique demonstrably successful in classification problems. In this context, SVMs establish a hyperplane, a linear decision boundary in lower dimensions or a higher-dimensional analogue, to optimally differentiate between two distinct classes within a dataset^37^. In SVMs, the goal is to create a hyperplane that maximizes the margin. This margin refers to the distance between the hyperplane and the nearest data points from individual class, called support vectors^38^. The primary objective of a linear SVM is to determine the most suitable separation boundary that optimizes the margin between two distinct classes. The determination of this border is contingent upon the location of the SV, where the data points that have the most influence on the classification process. When presented with a new data point, the SVM classifies it based on its location relative to the established hyperplane^39^.

For this study, the RBF kernel (Radial Basis Function) was used, as it is well-suited for datasets with non-linear relationships. However, the regularization parameter (C) and kernel parameter (gamma) were kept at default values due to the study’s focus on comparative evaluation rather than exhaustive hyperparameter tuning.

#### Decision tree

In the realm of injury severity analysis, DTs offer a robust approach to exploring the complex interplay between various factors and the resulting injury outcome. Unlike models that rely on specific assumptions about the data’s distribution, DTs thrive in uncovering non-linear relationships^40^. They achieve this by progressively dividing the data based on a series of yes/no questions (decision rules) applied to the different explanatory variables^41^. Each split aims to create the most distinct separation between data points belonging to different injury severity categories. The procedure persists until a predetermined termination point is attained, yielding a hierarchical arrangement resembling a tree, wherein each terminal node (leaf) corresponds to a distinct classification of damage severity^42,43^.

#### Random forest

RF leverages the power of ensemble learning for prediction. It constructs a collection of DTs using the training data. These trees capture the connections between the response variable (injury severity) and the independent variables (explanatory factors) through a tree-based approach. During prediction, the final prediction is the averaged output of individual trees. Notably, the training process incorporates randomness through random feature selection at each split, ensuring that individual trees do not rely too heavily on dominant variables. Instead of considering all available features at every decision point, RF selects a random subset of features, allowing for greater model diversity and reducing overfitting^44,45^. This approach enhances the generalization of the model, particularly in datasets with limited feature space, as in this study. This strategy offers several advantages. First, RF minimizes the need for manual parameter tuning, reducing the complexity of model development. Second, it demonstrates resilience to noise within the data, improving overall robustness. Finally, the ensemble approach effectively mitigates the risk of overfitting, leading to better prediction accuracy^20,46^.

#### Gradient boosting and extreme gradient boosting

In the quest to predict construction fatalities, GB and XGB emerge as powerful tools^47^. GB builds a sequence of DTs iteratively. Each new tree aims to improve upon the predictions of the previous one by focusing on the errors made. It starts with a baseline prediction and refines it by learning from the residuals (differences between actual and predicted values). These residuals are tackled by weak learners in the form of DTs, and the final prediction combines the initial estimate with the sum of all tree predictions, weighted by a learning rate. While adept at capturing complex patterns, careful hyperparameter tuning is crucial to avoid overfitting^48^. XGB takes Gradient Boosting a step further, employing more efficient algorithms for building DTs and incorporating regularization techniques to prevent overfitting. This makes it particularly effective for handling large datasets and complex relationships. Additionally, XGB boasts faster training times due to parallel processing capabilities and offers functionalities like tree pruning and missing value handling. XGB has gained popularity as a favoured method for addressing complex prediction tasks in the field of construction safety due to its exceptional execution and scalability. However, like GB, XGB requires appropriate hyperparameter adjustment to achieve the best outcomes^49–51^.

### Model explainability with SHAP

Model explainability was addressed using SHAP (SHapley Additive exPlanations), a game-theoretic approach that attributes each prediction to additive feature contributions under principled properties such as local accuracy and consistency^31,52^. For tree-based models (DT, RF, GB, XGB), the tree-specific SHAP formulation enables efficient, exact attributions while preserving these properties and supporting both instance-level and aggregate (global) interpretation^53^. More generally, SHAP connects to established individualized attribution methods grounded in Shapley values from cooperative game theory and has been shown to provide faithful explanations for a wide range of predictive models^54^. In this study, SHAP was used to quantify the relative influence of each input on predicted outcomes and to summarize feature contributions across the dataset, supporting transparent interpretation of the learned models.

### Evaluation parameters

In this study, the predictive performance of the ML models for SOI and NOI was quantified using six statistical evaluation metrics. These metrics were categorized into two groups, dissimilarity-based metrics (MSE and MAPE), which measure the error or deviation between predicted and observed values, and similarity-based metrics (R^2^, Precision, Recall, and F1-Score), which assess the agreement between predicted outcomes and actual incident severity^55,56^. The regression-oriented metrics (R^2^, MSE, MAPE) evaluate how closely the predicted severity scores align with the observed values, whereas the classification metrics (Precision, Recall, F1-Score) assess the models’ ability to distinguish severity levels and correctly identify high-risk incidents. The mathematical definitions, categories, and concise interpretations of each metric are presented in Table 2.

**Table 4:** Evaluation metrics for the predictive models.

Metric	Category	Formula	Remarks
R^2^	Regression, similarity-based	$\:1-\frac{\sum\:_{i=1}^{n}{{(y}_{i}-{\widehat{y}}_{i})}^{2}}{\sum\:_{i=1}^{n}{{(y}_{i}-{\stackrel{-}{y}}_{i})}^{2}}$	$\:0\le\:{R}^{2}\le\:1$, Higher values indicate better fit.
MSE	Regression, dissimilarity-based	$\:\frac{1}{n}\sum\:_{i=1}^{n}{{(y}_{i}-{\widehat{y}}_{i})}^{2}$	Lower values indicate better model performance.
MAPE		$\:\frac{1}{n}\sum\:_{i=1}^{n}\left|\frac{{(y}_{i}-{\widehat{y}}_{i}}{{y}_{i}}\right|\times\:100$	Lower values indicate lower relative error.
Precision	Classification, similarity-based	$\:\frac{TP}{TP+FP}$	Higher values mean fewer false positives.
Recall		$\:\frac{TP}{TP+FN}$	Higher values mean fewer missed severe cases.
F1-Score		$\:2\times\:\frac{Precision\:\times\:Recall}{Precision+Recall}$	Higher values reflect balanced precision and recall.

Note: $\:{y}_{i}$ and $\:{\widehat{y}}_{i}$ denote the observed and predicted severity values for the $\:i$*-*th incident, $\:\stackrel{\prime }{y}$ is the mean of the observed values, and $\:n$ is the number of samples*.*$\:TP$, $\:TN$, $\:FP$, and $\:FN$ represent true positives, true negatives, false positives, and false negatives, respectively*.*..

## Results and discussion

This section details the model implementation, validation, predictive performance, and SHAP-based interpretation by several established ML models.

### Model implementation

Models were trained on the SOI and NOI dataset using an 80/20 train-test split with a fixed random state of 123. For multi-class classification tasks (NOI and SOI), splits were stratified by the target variable to preserve class proportions. Categorical variables were encoded as described in Sect. “Random forest”. Feature standardization (mean = 0, standard deviation = 1) was applied only to SVM and KNN, while tree-based and boosting algorithms were trained on raw numeric inputs. To mitigate class imbalance, SMOTE was applied exclusively to the training set, leaving the test set untouched. In addition, when supported, class weights were set inversely proportional to class frequency. Each model was trained once on the training data and subsequently evaluated on the held-out test data. Table 4 summarizes the estimators, tasks, and hyperparameters used for model implementation.

**Table 5:** Model implementation details, including training settings and hyperparameters used for training.

Model	Key training settings	Core hyperparameters
**KNN**	Standardized features; stratified 80/20 split; SMOTE on training set only	n_neighbors = 5, weights=’uniform’, metric=’minkowski’, *p* = 2
**SVM**	Standardized features; probability = True for ROC; stratified 80/20 split; SMOTE on training set only	kernel=’rbf’, C = 1.0, gamma=’scale’
**DT**	No scaling required; stratified 80/20 split	criterion=’gini’, max_depth = None, min_samples_split = 2, min_samples_leaf = 1, random_state = 123
**RF**	No scaling required; stratified 80/20 split	n_estimators = 100, criterion=’gini’, max_features=’sqrt’, bootstrap = True, random_state = 123
**GB**	No scaling required; class weights inverse to frequency (where supported); stratified 80/20 split	n_estimators = 100, learning_rate = 0.1, max_depth = 3, subsample = 1.0, random_state = 123
**XGB**	No scaling required; stratified 80/20 split; SMOTE on training set only.	n_estimators = 100, learning_rate = 0.3, max_depth = 6, subsample = 0.8, colsample_bytree = 0.7, eval_metric = rmse/mlogloss, random_state = 123

### Model performance and evaluation

#### Model validation

The study compared six models: SVM, KNN, DT, RF, GB, and XGB. Overall, boosting and bagging models demonstrated superior performance compared to linear models. Notably, XGB achieved the highest accuracy, outperforming all other models in both NOI and SOI predictions.

**Fig. 3:**
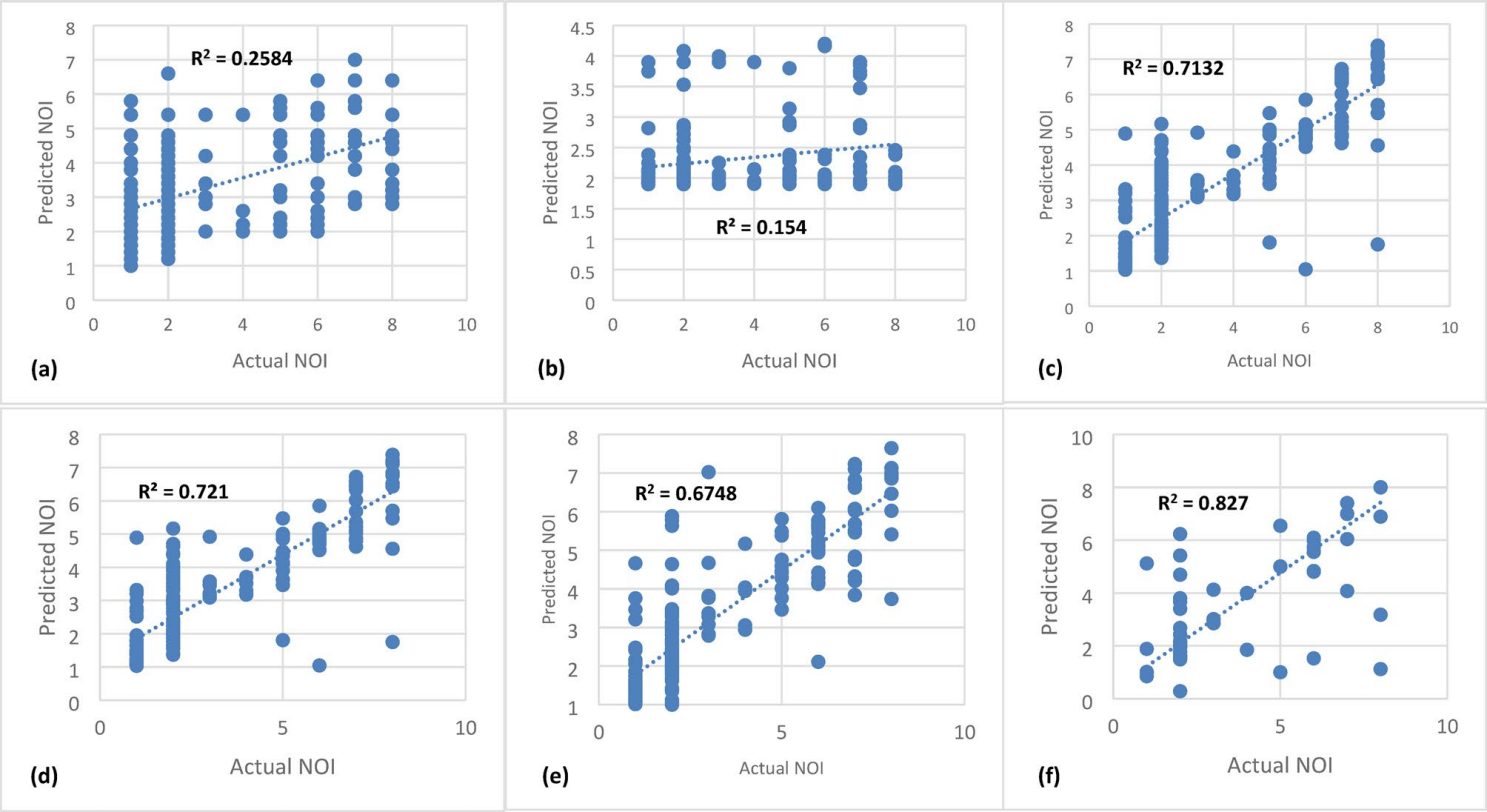
Nature of incident prediction (X1-X14): **(a)** KNN, **(b)** SVM, **(c)** DT, **(d)** RF, **(e)** GB, **(f)** XGB.

**Fig. 4:**
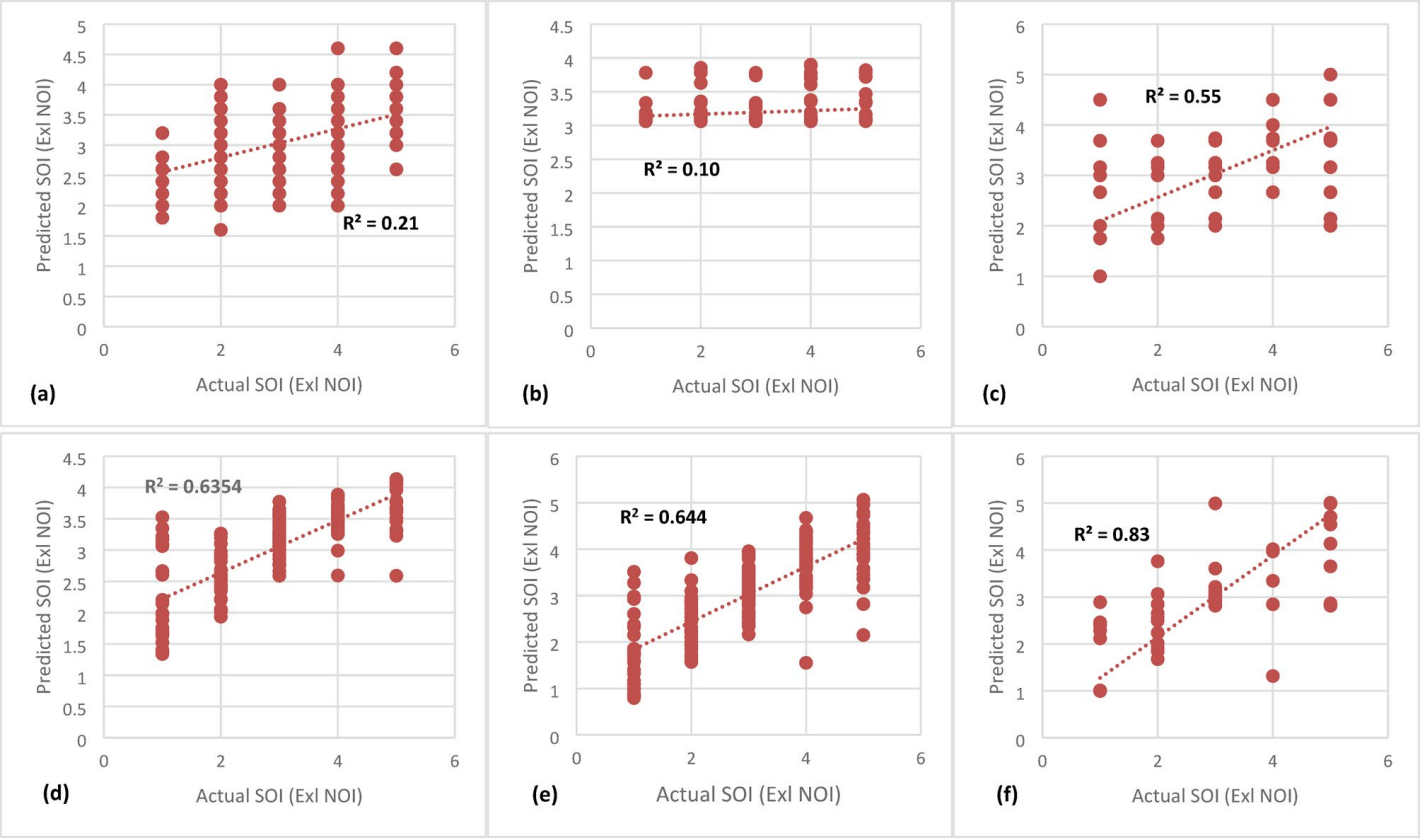
Severity level of Incident Prediction (X1-X14): **(a)** KNN, **(b)** SVM, **(c)** DT, **(d)** RF, **(e)** GB, **(f)** XGB.

For NOI prediction (Fig. 3), XGB achieved an accuracy of 82%, followed by RF (72%) and DT (71%), confirming the advantage of ensemble models over individual DTs. Similarly, for SOI prediction (Fig. 4), the top-performing models remained XGB (83%), RF (64%), and GB (65%), reinforcing the effectiveness of boosting techniques.

**Fig. 5:**
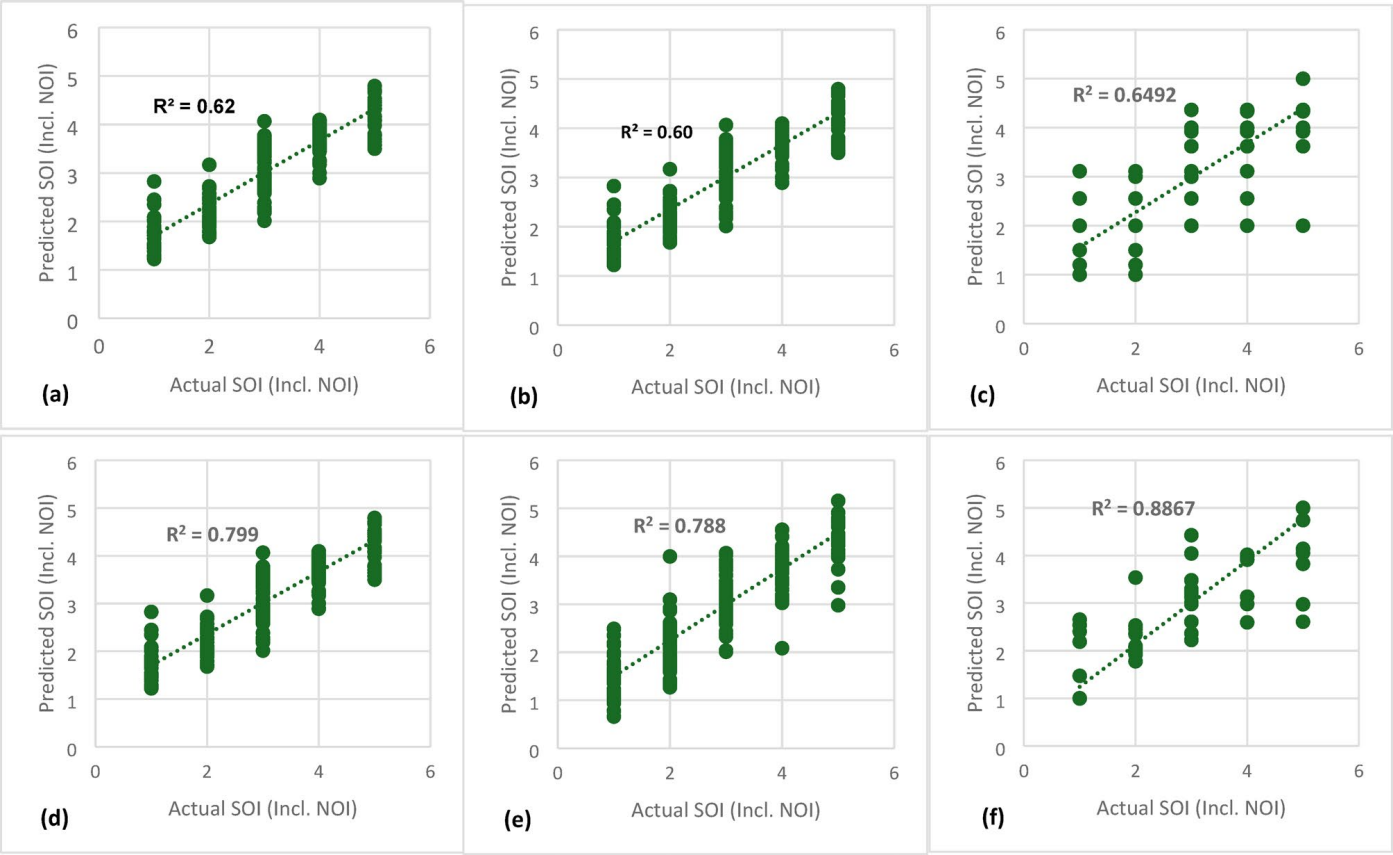
Severity level of Incident Prediction (X1-X14, NOI): **(a)** KNN, **(b)** SVM, **(c)** DT, **(d)** RF, **(e)** GB, **(f)** XGB.

Given the potential causal relationship between NOI and SOI, an additional feature (NOI) was incorporated to enhance SOI prediction (Fig. 5). In this scenario, XGB remained the most effective model, achieving an accuracy of 89% when using X1-X14 and NOI as input variables. RF and GB followed closely, with 80% and 78% accuracy, respectively. In contrast, SVM and KNN performed poorly (< 26%) in both NOI and SOI prediction, indicating that linear models struggle with complex severity classification. These results align with findings from prior studies^16,23^, which also highlighted the limitations of linear models in non-linear, high-dimensional safety prediction tasks. Table 5 shows R^2^, MSE and MAPE% of all models employed in this study.

**Table 6:** Evaluation parameters (R^2^, MSE, MAPE%) of established models.

Models	NOI (excluding SOI)			SOI (excluding NOI)			SOI (including NOI)		
	*R* ^2^	MSE	MAPE %	*R* ^2^	MSE	MAPE %	*R* ^2^	MSE	MAPE %
KNN	0.26	2.98	1.38	0.21	3.05	1.43	0.62	2.48	0.90
SVM	0.15	4.15	1.67	0.10	4.28	1.82	0.60	2.54	0.92
DT	0.71	1.83	0.47	0.55	2.34	0.83	0.65	2.01	0.59
RF	0.72	1.84	0.48	0.64	2.06	0.58	0.80	0.95	0.38
GB	0.68	2.03	0.55	0.64	2.05	0.56	0.78	1.56	0.40
XGB	0.82	1.08	0.35	0.83	1.03	0.34	0.89	0.82	0.31

#### Precision, recall, and F1-score analysis

In multiclass classification problems, accuracy alone can be misleading, as some models may favour certain classes while neglecting others. To ensure a more balanced evaluation, precision, recall, and F1-score were also calculated to provide deeper insight into model performance for SOI (including NOI). These metrics offer a more comprehensive view of model effectiveness, particularly when handling imbalanced severity classes.

The results in Table 6 indicate that XGB achieved the highest recall (0.707) and F1-score (0.691), confirming its strong ability to identify severe incidents while maintaining precision. GB followed closely, reinforcing the advantage of boosting models in improving classification performance. The RF model exhibited strong precision (0.687) but had a lower recall (0.585), indicating a conservative prediction approach. It correctly classified most of the severe cases it identified but failed to detect some high-risk events. DT performed moderately well, maintaining a balanced precision-recall trade-off but was less effective than RF or boosting models.

Conversely, KNN and SVM showed the lowest recall scores, signifying that they failed to detect a substantial number of severe incidents. SVM had the lowest overall F1-score (0.187), highlighting its high misclassification rates. These results confirm that SVM and KNN are unsuitable models for severity classification due to their inability to generalize well to complex incident patterns.

Table 7, which focuses exclusively on SOI Level 5 (fatal incidents), provides deeper insights into the models’ ability to predict the most critical severity class. XGB again outperformed the other models, achieving the highest F1-score (0.700) and strong precision (0.777), underscoring its ability to identify fatal incidents effectively. GB and RF also demonstrated robust performance, with F1-scores of 0.695 and 0.692, respectively. However, DT, KNN, and particularly SVM struggled to perform in this category, with SVM achieving the lowest F1-score (0.214). These findings align with the overall evaluation in Table 5, further validating the reliability of boosting-based models like XGB and GB for accurately identifying high-risk events in construction safety (Table 8).

**Table 7:** Evaluation parameters (Precision, Recall, F1 Score) of established models in predicting SOI (Level 1–5).

Models	SOI (including NOI) – Level 1–5		
	Precision	Recall	F1 Score
KNN	0.483	0.463	0.439
SVM	0.583	0.268	0.187
DT	0.647	0.658	0.641
RF	0.687	0.585	0.560
GB	0.731	0.682	0.668
XGB	0.733	0.707	0.691

**Table 8:** Evaluation parameters (Precision, Recall, F1 Score) of established models in predicting SOI (Level 5 only).

Models	SOI (including NOI) – Level 5 only		
	Precision	Recall	F1 Score
KNN	0.440	0.363	0.400
SVM	0.176	0.272	0.214
DT	0.500	0.250	0.333
RF	0.600	0.818	0.692
GB	0.666	0.727	0.695
XGB	0.777	0.636	0.700

#### Receiver operating characteristic (ROC) curve analysis

The Receiver Operating Characteristic (ROC) curve is a graphical representation that evaluates the ability of ML models to distinguish between different severity levels of incidents. It plots the True Positive Rate (TPR) against the False Positive Rate (FPR) across various classification thresholds, providing an insightful measure of model discrimination performance. The area under the ROC curve (AUC-ROC) is a critical metric that quantifies the model’s ability to correctly classify incidents at different severity levels, with values closer to 1.0 indicating superior performance.

In this study, multiclass ROC curves were generated using the One-vs-Rest (OvR) approach, where the classification performance for each severity level was assessed separately (Fig. 5(a-f)). To provide a more thorough evaluation, Micro-Average AUC scores were used to compare models, as they aggregate performance across all severity levels.

The results indicate a clear distinction between the performance of boosting-based models (XGB and GB) and traditional ML models (RF, DT, SVM, and KNN). Among the tested models, XGB achieved the highest Micro-Average AUC score of 0.89, demonstrating its ability to consistently distinguish between severity levels. GB followed closely with a Micro-Average AUC of 0.86, reinforcing the effectiveness of boosting-based techniques in handling complex classification tasks.

Conversely, RF and DT exhibited moderate performance, with RF achieving a Micro-Average AUC of 0.80 and DT scoring 0.79. While RF benefited from its ensemble learning capability, it still struggled in cases where severity levels overlapped. The lowest Micro-Average AUC scores were observed for KNN (0.68) and SVM (0.62), suggesting that these models struggled to effectively differentiate between different severity levels.

#### Confusion matrix heatmap analysis

**Fig. 6:**
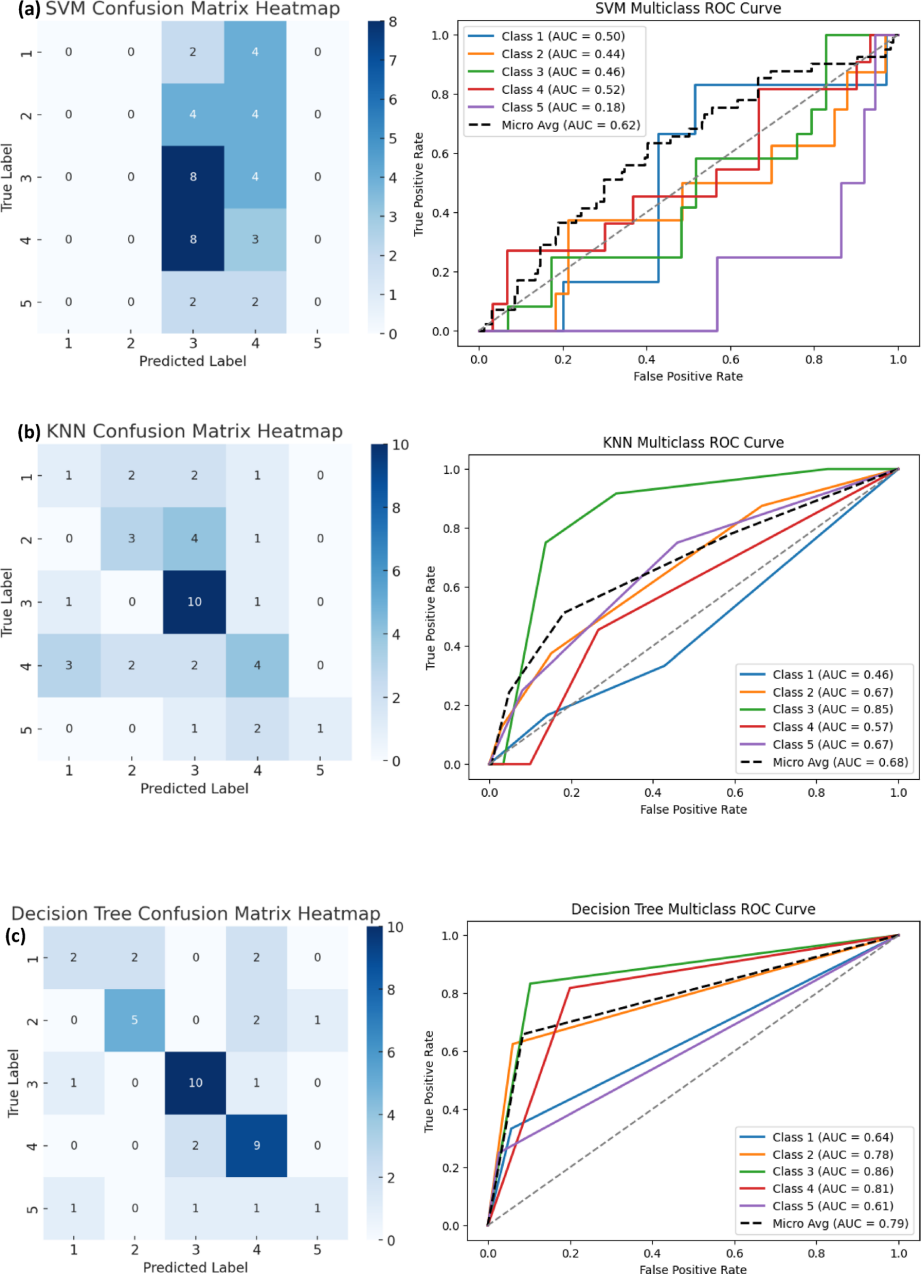

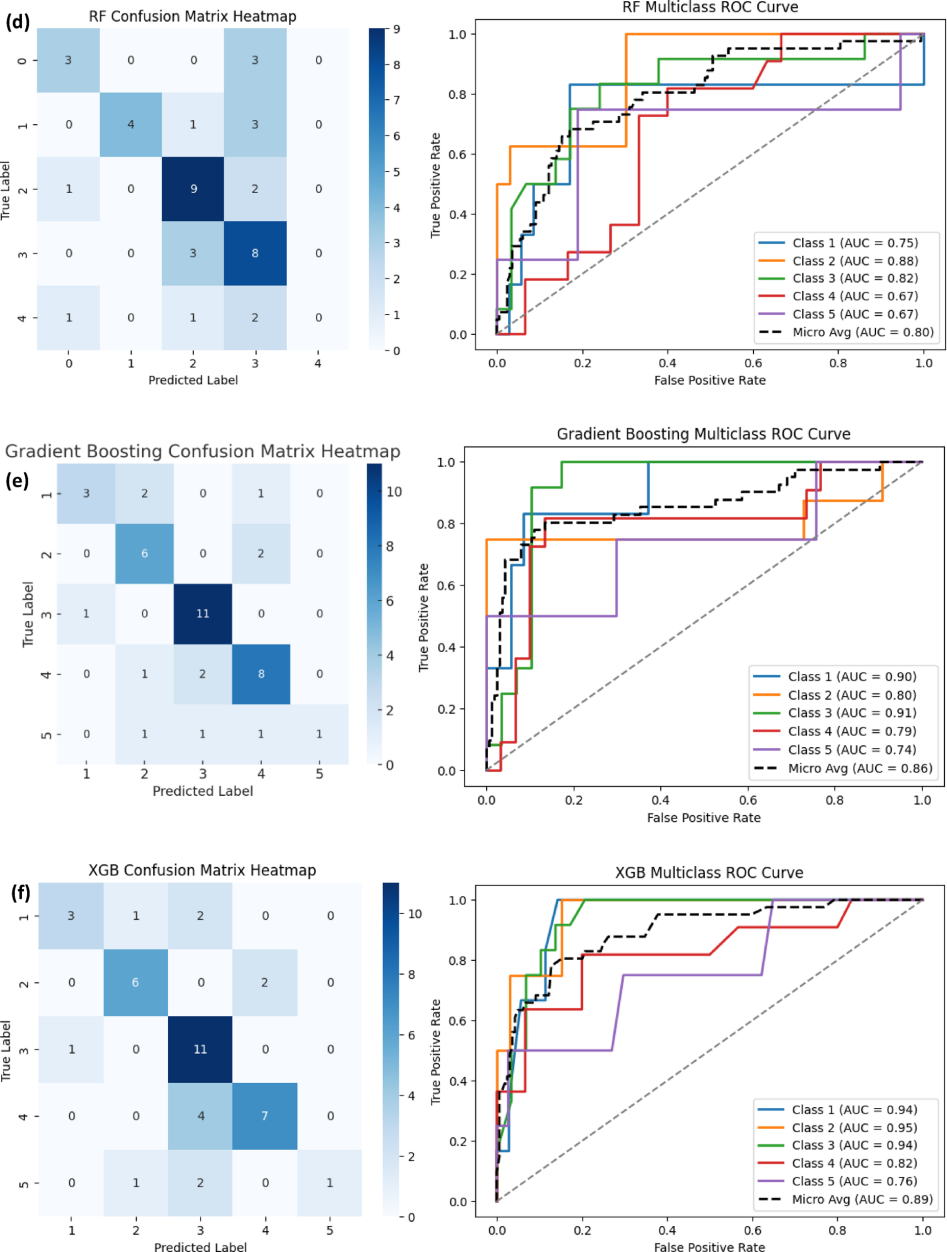
Confusion matrix heatmap and ROC curve of ML models: **(a)** SVM, **(b)** KNN, **(c)** DT, **(d)** RF, **(e)** GB, **(f)** XGB.

To further understand the model performance, confusion matrices were generated for each ML model to visualize how accurately they classified different severity levels. The confusion matrix provides a detailed breakdown of correct and incorrect predictions, helping to identify trends in misclassification across different severity categories (Figure. 6(a-f)).

The XGB and GB models demonstrated the highest accuracy, with fewer misclassifications across severity levels. These models showed strong predictive capability for high-severity incidents (Severity Level 5), minimizing errors in distinguishing critical safety events. This result aligns with the high AUC scores observed in the ROC analysis, confirming that boosting-based models are the most reliable for severity classification.

The RF model also showed competitive performance, but it had slightly higher misclassification rates between severity levels 3 and 4. This suggests that while RF is a powerful ensemble model, it struggles in differentiating medium-to-high severity cases due to overlapping incident characteristics. The DT model exhibited similar performance but with higher misclassification rates compared to RF, reaffirming the benefit of ensemble techniques.

Conversely, KNN and SVM performed poorly in distinguishing high-severity incidents, with significant misclassification between severity levels 3, 4, and 5. SVM, in particular, failed to capture severe incidents, frequently misclassifying high-risk cases as lower-severity incidents. This aligns with its low recall and F1-score, suggesting that SVM is not suitable for safety incident severity classification.

The results of this study highlight that boosting-based models, particularly XGB and GB, outperformed all other models in terms of evaluation parameters, accuracy, AUC, confusion matrix accuracy, making them the most effective for predicting safety incident severity in construction projects. Among the evaluated models, RF also demonstrated strong performance, particularly in precision-based evaluation. However, RF was less reliable than XGB in capturing high-risk incidents such as fatalities (SOI Level 5). DT exhibited moderate performance but lacked the robustness offered by ensemble methods. In contrast, KNN and SVM were the least effective, struggling to differentiate between severity levels due to their inability to capture non-linear relationships and complex decision boundaries.

The dataset contains non-linear relationships and interactions between explanatory variables (e.g., the combined effect of PPE usage, safety training, and incident location), which are better captured by ensemble methods. Models like RF and XGB aggregate multiple DTs, each specializing in different regions of the feature space, allowing them to handle complex decision boundaries effectively. Additionally, ensemble methods are inherently robust to noise and high-dimensional interactions due to their use of bagging (in RF) and boosting (in XGB). Boosting algorithms, for example, iteratively refine the model by focusing on hard-to-classify instances, ensuring improved performance on imbalanced and noisy datasets.

In contrast, linear models like SVM and KNN rely on simpler decision boundaries and often struggle with datasets exhibiting significant non-linearity or high-dimensional feature interactions. For instance, SVM assumes a fixed kernel or linear separability, which limits its ability to capture complex patterns in the data. The RBF kernel was used to address non-linearity, but the lack of exhaustive hyperparameter tuning (e.g., the regularization parameter C and kernel parameter gamma) constrained performance. In addition, SVM is sensitive to class imbalance and often misclassifies minority class instances, such as SOI Level 5, due to their underrepresentation. Similarly, KNN, which relies on local neighbourhoods for classification, is particularly affected by the curse of dimensionality in high-dimensional datasets. The use of Euclidean distance as the similarity measure becomes less effective when the number of features increases, leading to poorer discrimination between classes. Furthermore, the choice of k = 5 limited the model’s ability to adapt to the dataset’s complexities, especially in handling imbalanced classes where minority instances are underrepresented in local neighbourhoods.

These findings suggest that advanced ensemble techniques like boosting significantly improve safety prediction accuracy, offering practical applications for risk mitigation and incident prevention in the construction industry. By leveraging these models, construction safety assessment systems can proactively enhance decision-making, reducing the likelihood of severe incidents. Given the results, XGB is recommended as the most suitable model for implementing proactive safety assessment systems, given its superior performance in distinguishing incident severity and handling complex datasets.

### Feature importance and SHAP interpretability

**Fig. 7:**
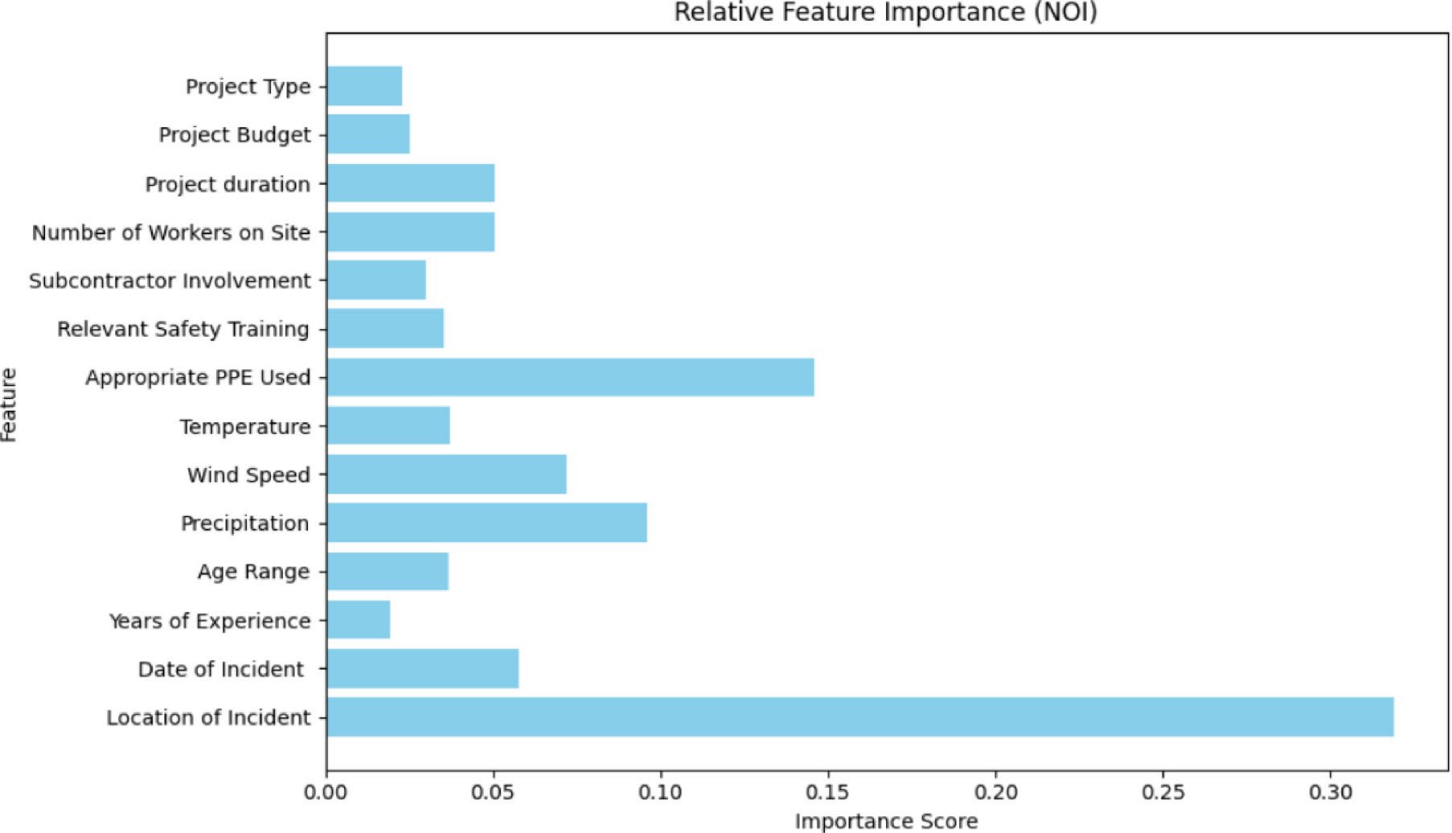
Feature importance of input variables in predicting the NOI.

The proportional importance of input variables offers insight into the drivers of prediction across tasks. For forecasting the NOI (Fig. 7), the Location of Incident emerged as the most critical factor (≈ 0.35), followed by Appropriate PPE Use (≈ 0.15), Precipitation (≈ 0.08), and Wind Speed (≈ 0.07). These results highlight actionable priorities, as incidents occurring in scaffolding areas or in locations with poor PPE compliance are consistently linked with more severe outcomes. Such evidence supports targeted safety audits for high-risk sites and stricter enforcement of PPE policies.

**Fig. 8:**
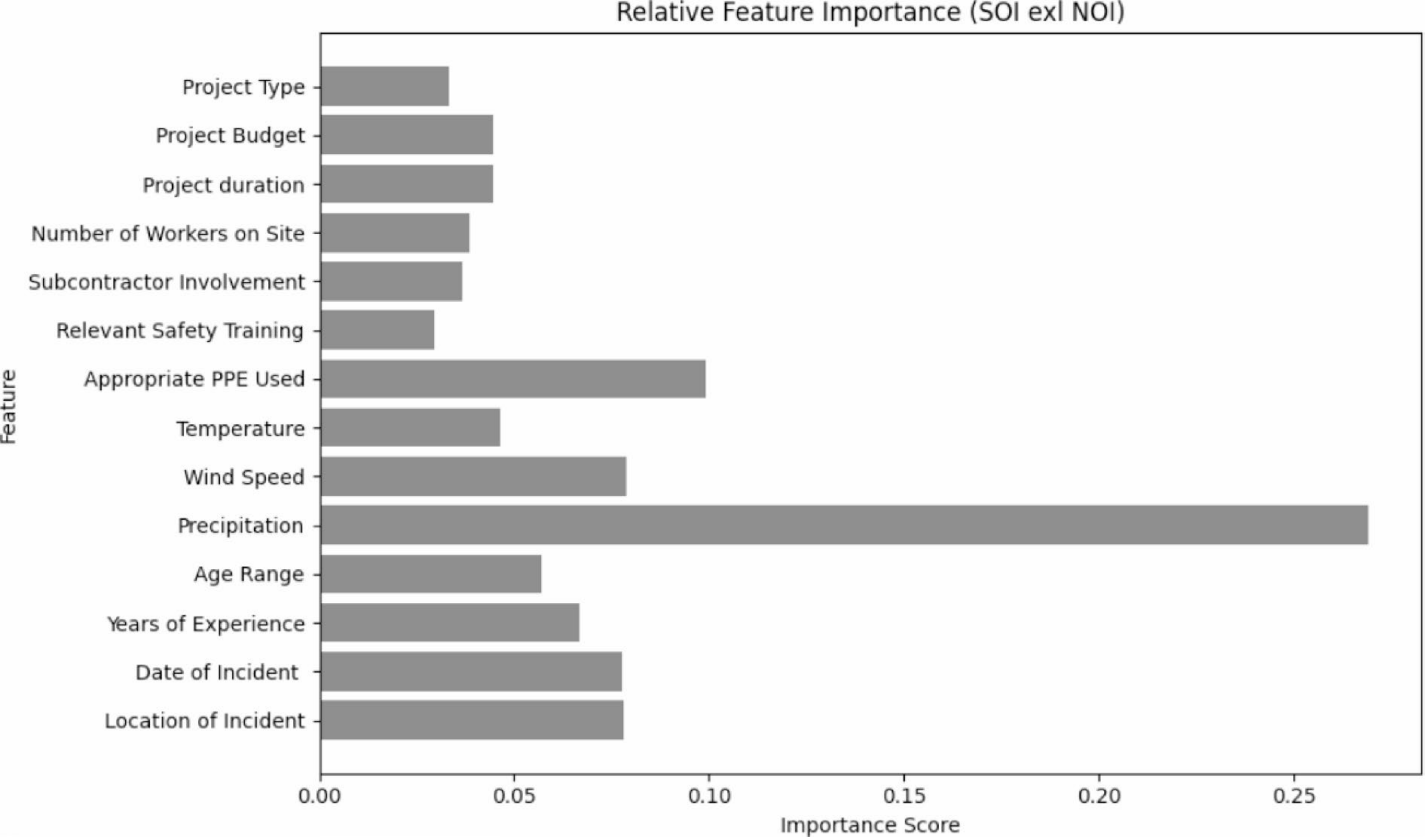
Feature importance of input variables in predicting the SOI (X1-X14).

For SOI prediction without NOI (Fig. 8), Precipitation dominated importance (≈ 0.30), followed by Appropriate PPE Use (≈ 0.10) and Location of Incident (≈ 0.08). The strong role of precipitation indicates that adverse weather conditions substantially affect severity outcomes, as heavy rainfall can compromise visibility, increase slip risks, and weaken scaffolding stability. These findings underscore the need for weather-adaptive safety planning, including schedule adjustments, drainage improvements, and workforce training for hazardous weather.

**Fig. 9:**
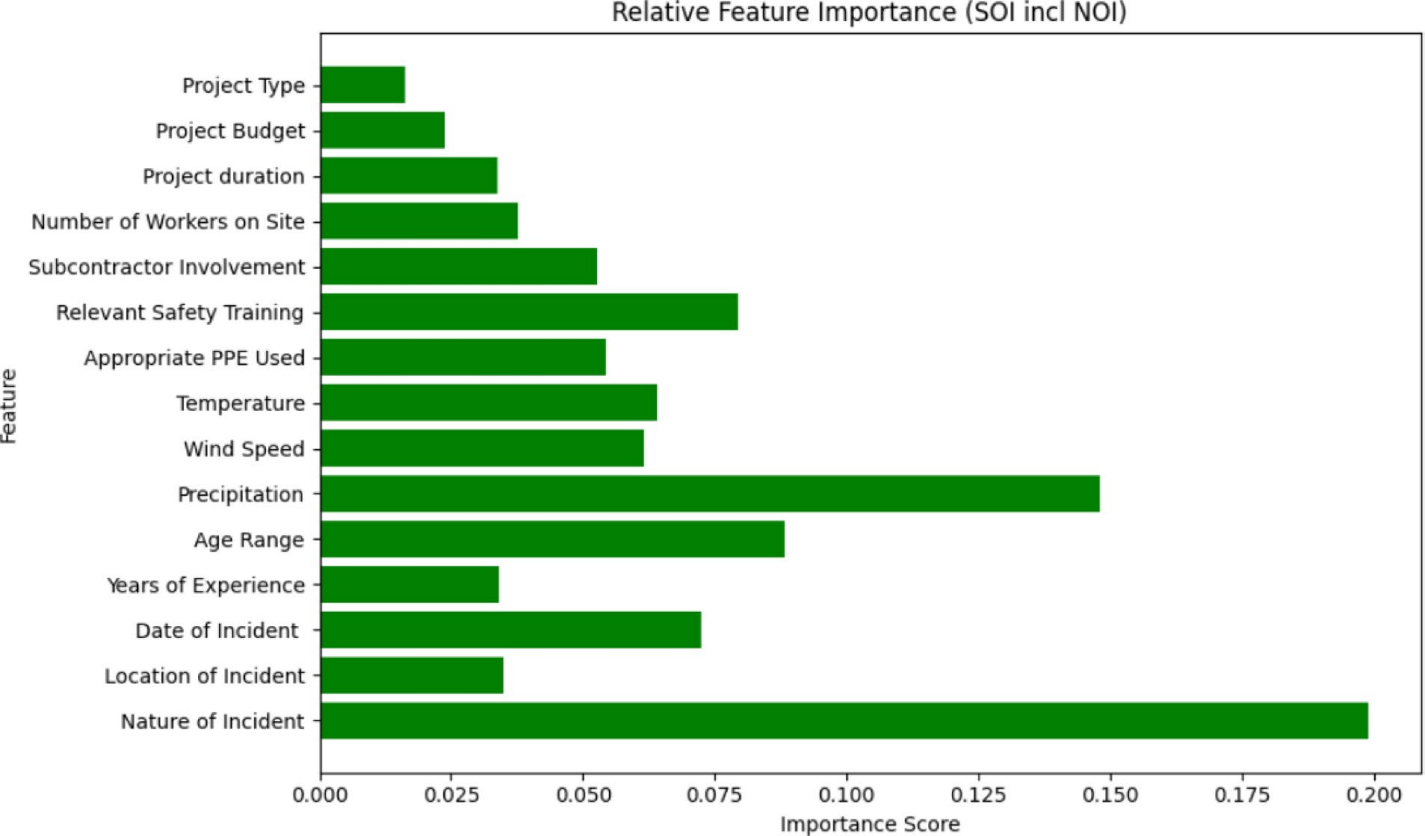
Feature importance of input variables in predicting the SOI (X1-X14, NOI).

When NOI is included in SOI prediction (Fig. 9), the NOI became the most significant factor (≈ 0.20), with severe types such as falls, traffic accidents, and electrical shocks contributing most strongly to higher fatality risks. Precipitation (≈ 0.15) retained influence, while Age Range (≈ 0.08), Relevant Safety Training (≈ 0.075), and Appropriate PPE Use (≈ 0.05) also featured prominently. These results emphasize that severity is shaped by the interaction of incident type, environmental exposure, and organizational practices. Collectively, the feature importance patterns explain the marked improvement in XGB performance when NOI is incorporated, as it captures critical distinctions among incident types that strongly determine severity.

**Fig. 10:**
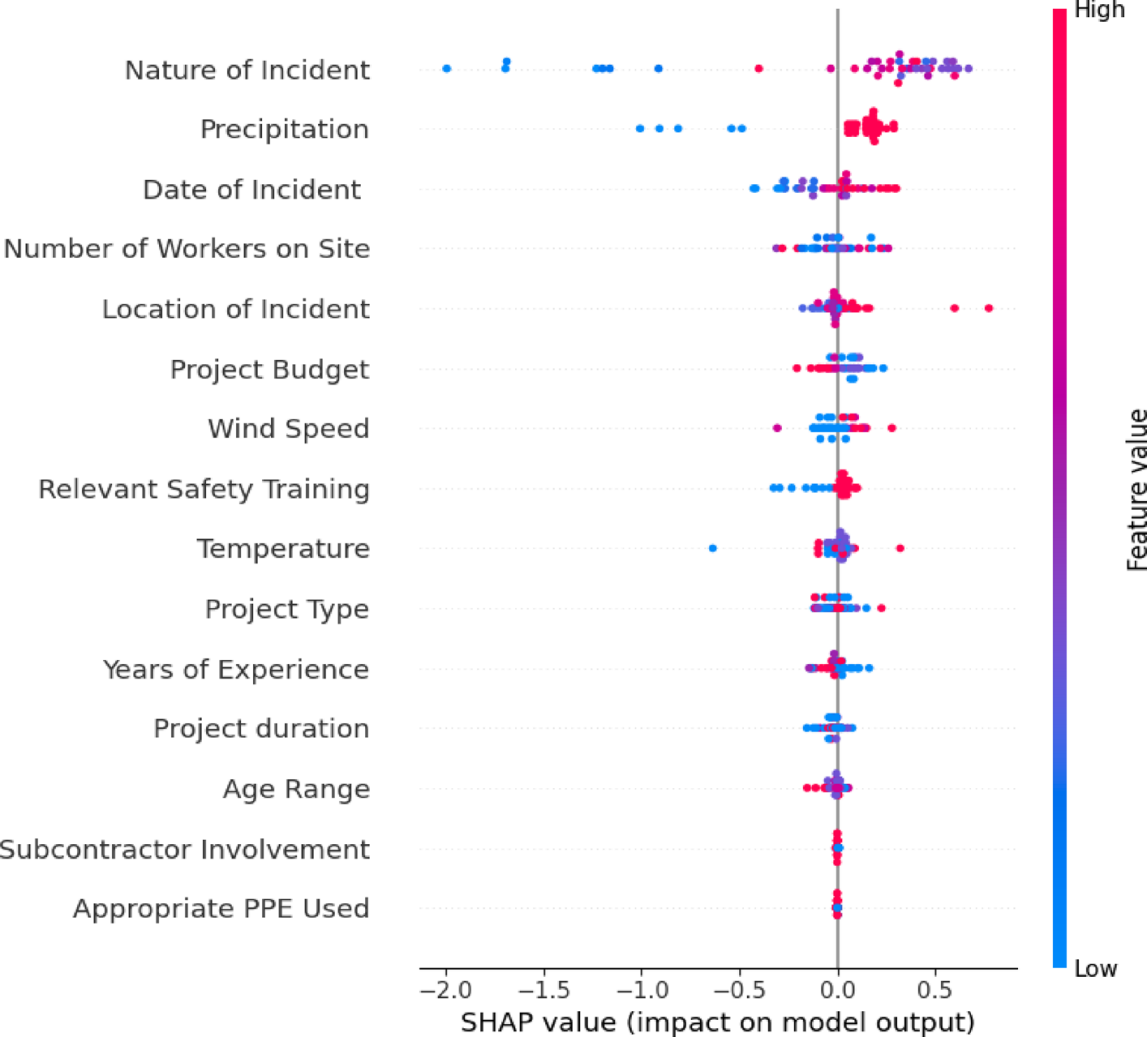
SHAP summary plot for feature contributions to SOI predictions.

To complement these rankings, SHAP analysis was performed to enhance the interpretability of the ML models and quantify the influence of individual features on the prediction of safety incident severity. SHAP values provide a robust method to determine the impact of each feature on the model’s output, ensuring transparency and actionable insights for stakeholders^31,52^. Figure 10 presents the SHAP summary plot, ranking features by their average absolute SHAP values, with higher values indicating greater influence.

The analysis identifies “Nature of Incident,” “Precipitation,” and “Date of Incident” as the most influential features, followed by “Number of Workers on Site” and “Location of Incident.” Among these, “Nature of Incident” had the highest impact, emphasizing its role in determining safety outcomes. Severe incident types, such as falls from height or collisions, had a substantial effect on model predictions, underscoring the importance of proactive safety interventions. Precipitation also emerged as a significant factor, as it increases slip hazards and reduces visibility, highlighting the need for weather-adaptive safety measures. Additionally, the “Date of Incident” revealed temporal patterns, with certain months exhibiting higher risks, providing opportunities for seasonal safety planning. Similarly, proper PPE usage and Relevant Safety Training positively contribute to mitigating incident severity, emphasizing the actionable insights these features provide for safety interventions.

Taken together, these SHAP results align with the feature importance graphs (Figs. 7, 8 and 9), which also identify NOI, precipitation, and PPE use as pivotal factors influencing SOI predictions. The inclusion of NOI as a feature not only enhances interpretability but also explains the substantial increase in XGB’s SOI prediction accuracy (89%) when it is incorporated. This improvement demonstrates the critical role of NOI in the model’s predictive power and supports the study’s findings on the strong connection between SOI and NOI. Moreover, this analysis supports the prioritization of safety interventions, such as targeted training programs based on the most influential incident factors, enhanced monitoring of high-risk areas, and the integration of weather forecasts into daily site planning. Overall, SHAP analysis strengthens the interpretability of the model, providing construction managers and safety officers with clear guidance for implementing effective safety measures.

## Practical Implications, future research directions and research limitations

### Practical implications

This study provides actionable insights into improving safety management practices in construction, particularly in safety-critical environments. The results highlight how ML models can be leveraged for risk assessment, proactive interventions, and resource allocation:

1. **Targeted Safety Interventions**
  The analysis identified high-risk areas (e.g., scaffolding zones) where incidents are more likely to occur, allowing site managers to implement stricter safety protocols.
  PPE compliance remains a key factor in reducing injury severity. Enhanced enforcement of PPE usage policies can significantly improve worker safety.
  Weather-related risks (precipitation, wind speed) should be integrated into daily construction site planning to mitigate incident risks.

2. **Practical Use of Models in Decision-Making**
  While SOI prediction achieved high accuracy (89%), NOI prediction (60%) requires careful interpretation.
  ML models should be used as decision-support tools rather than standalone predictive systems, guiding risk-based safety audits and resource prioritization.

## Adapting ML-based safety models for different contexts

While this study focused on the Saudi Arabian construction industry, its methodology can be adapted for other regions by incorporating local climate, workforce demographics, and regulatory frameworks.

Future work should explore region-specific variations in accident patterns to improve model applicability in diverse construction environments.

### Future research directions

Despite its contributions, this study has limitations that open pathways for future research:

1. **Enhancing Accuracy for NOI Prediction**
  Expand the dataset by incorporating additional sources of safety data, such as near-miss reports, worker behaviour records, and IoT-based monitoring systems.
  Apply advanced feature engineering, such as interaction terms and domain-specific variables, to better capture the relationships between factors affecting incident occurrence.
  Experiment with ensemble techniques like stacking and hyperparameter optimization to improve model robustness.

2. **Addressing Dataset Limitations**
  The dataset was limited to 203 incidents, which constrains generalizability. Future research should use larger, more diverse datasets to improve model performance.
  Some explanatory variables (e.g., time with employer, time of incident, specific PPE types) were excluded due to missing data. Collecting complete records could enhance model accuracy.

3. **Exploring Deep Learning and Evolutionary Algorithms**
  Deep learning models have the potential to process complex, high-dimensional safety data but require larger datasets and computational resources.
  Evolutionary algorithms could help optimize feature selection and enhance model efficiency, improving the accuracy of safety predictions.

4. **Adapting the Methodology for Broader Applications**
  The methodology should be validated in different countries and industries by adjusting feature selection based on region-specific safety regulations and workforce demographics.
  Applying the model to different construction sectors (residential, industrial, infrastructure) could improve its versatility in safety management.

5. **Comparative Studies with Statistical Models**
  While ML techniques outperformed traditional models, future studies should conduct a comparative analysis with statistical models, such as multinomial logit and probit models, to understand their relative strengths.
  This would help determine the best approach for different types of safety predictions, ensuring optimal decision-making in construction risk assessment.

### Research limitations

The dataset used in this study contains 203 construction-safety incidents collected over a six-year period from two regions in Saudi Arabia. The relatively small sample size and limited geographic coverage may reduce the ability to generalize the findings, especially for less common types and levels of incidents. Differences in how incidents were reported across projects, along with possible missing information, could introduce bias. In addition, the lack of a fully standardized format for recording incidents makes it difficult to compare variables consistently. Since the analysis is based on past administrative records, some important factors such as near-misses or detailed crew-level conditions may not have been recorded. Changes in safety regulations and site practices during the study period may also affect the consistency of the data. Finally, the models were not validated on data from other regions or organizations, which limits testing their transferability. These limitations highlight the need for future research to use larger, more diverse datasets, ideally collected under common standards and validated on independent samples.

## Conclusions

This study analyses 203 construction safety incidents from Saudi Arabia (primarily Makkah and Riyadh) to develop predictive models for both the NOI and the SOI using 14 explanatory variables. These variables encompass project type, budget, worker demographics (age and experience), and fatality-related information. The modelling is complemented by SHAP-based interpretability to identify the most influential factors for decision-making. The study has the following conclusions.

- All models achieved meaningful accuracy for both tasks; XGB performed best, reaching 89% accuracy for SOI, while NOI prediction exceeded 60% across models. Incorporating NOI as an input improved SOI prediction, underscoring the linkage between incident characteristics and severity.
- Boosting methods (XGB, GB) consistently outperformed non-boosting baselines, indicating advantages in capturing non-linearities and feature interactions present in safety data.
- Nature of incident, precipitation, location of incident, number of workers on site, and safety practices (PPE compliance, relevant training) emerged as the most influential determinants, with SHAP confirming their direction and relative impact.
- Findings support weather-adaptive planning (e.g., schedule adjustments during rainfall), targeted controls at high-risk locations (e.g., scaffolding/workshops), and reinforcement of PPE use and safety training. These actions can be embedded into site checklists, toolbox talks, and escalation protocols.

These results highlight the potential of ML and explainable analytics to support data-driven safety management. At the same time, the evidence should be interpreted considering the sample size and regional scope, and would benefit from external validation on larger, multi-region datasets curated under common taxonomies. Future work can extend this study by integrating narrative text (e.g., incident descriptions) and real-time weather feeds, exploring ordinal/cost-sensitive objectives for severity, and evaluating deployment aspects (alert thresholds, periodic model recalibration, and fairness checks). Collectively, the contributions point toward practical, explainable tools that can help organizations anticipate risk and prioritize preventive interventions on site.

## Data Availability

The data is available from the corresponding author upon request.

## References

[1] Home | U.S. Department of Labor. (n.d.). accessed March 9, (2024). https://www.dol.gov/

[2] Statistics -. Work-related fatal injuries in Great Britain, (n.d.). accessed March 9, (2024). https://www.hse.gov.uk/statistics/fatals.htm

[3] Gholamizadeh, K., Tapak, L., Mohammadfam, I. & Soltanzadeh, A. Investigating the work-related accidents in iran: analyzing and comparing the factors associated with the duration of absence from work. *Iran. Rehabilitation J.* **20**, 589–600. 10.32598/IRJ.20.4.1739.1 (2022).

[4] Moradinazar, M., Kurd, N., Farhadi, R., Amee, V. & Najafi, F. Epidemiology of work-related injuries among construction workers of Ilam (Western iran) during 2006–2009. *Iran. Red Crescent Med. J.* **15**10.5812/IRCMJ.8011 (2013). 10.5812/ircmj.8011 PMC3950784 24693372

[5] Hinze, J. & Wilson, G. Moving toward a Zero Injury Objective, *J. Constr. Eng. Manag.* **126**, 399–403. (2000). 10.1061/(ASCE)0733-9364(2000)126:5(399).

[6] Kineber, A. F. et al. Revolutionizing construction: A cutting-edge decision-making model for artificial intelligence implementation in sustainable Building projects. *Heliyon* **10**, e37078. 10.1016/J.HELIYON.2024.E37078 (2024). 39286223 10.1016/j.heliyon.2024.e37078 PMC11402912

[7] Abioye, S. O. et al. Artificial intelligence in the construction industry: A review of present status, opportunities and future challenges. *J. Building Eng.* **44**, 103299. 10.1016/J.JOBE.2021.103299 (2021).

[8] Alam Bhuiyan, M. M. & Hammad, A. A hybrid multi-criteria decision support system for selecting the most sustainable structural material for a multistory building construction, *Sustainability,* **15**, 3128 15. (2023). 10.3390/SU15043128

[9] Waqar, A. et al. Success of Implementing Cloud Computing for Smart Development in Small Construction Projects, *Applied Sciences ***13**, 5713 13. (2023). 10.3390/APP13095713

[10] Fazil, A. Z., Gomes, P. I. A. & Sandamal, R. M. K. Applicability of machine learning techniques to analyze microplastic transportation in open channels with different hydro-environmental factors. *Environ. Pollut*. **357**10.1016/J.ENVPOL.2024.124389 (2024). 10.1016/j.envpol.2024.124389 38906408

[11] Zhong, B., Pan, X., Love, P. E. D., Sun, J. & Tao, C. Hazard analysis: A deep learning and text mining framework for accident prevention. *Adv. Eng. Inform.* **46**, 101152. 10.1016/J.AEI.2020.101152 (2020).

[12] Kannan, R., Abdul Halim, H. A., Ramakrishnan, K., Ismail, S. & Wijaya, D. R. Machine learning approach for predicting production delays: a quarry company case study. *J. Big Data*. **9**, 1–12. 10.1186/S40537-022-00644-W/TABLES/3 (2022). 10.1186/s40537-022-00644-w PMC9287717 35875725

[13] Samy, S. S. et al. Adoption of machine learning algorithm for predicting the length of stay of patients (construction workers) during COVID pandemic. *Int. J. Inform. Technol. (Singapore)*. **15**, 2613–2621. 10.1007/S41870-023-01296-6/FIGURES/15 (2023). 10.1007/s41870-023-01296-6 PMC10250170 37360312

[14] Bzdok, D. Classical statistics and statistical learning in imaging neuroscience. *Front. Neurosci.* **11**10.3389/FNINS.2017.00543 (2017). 10.3389/fnins.2017.00543 PMC5635056 29056896

[15] Bzdok, D., Altman, N. & Krzywinski, M. Points of significance: statistics versus machine learning. *Nat. Methods*. **15**, 233–234. 10.1038/NMETH.4642 (2018). 30100822 10.1038/nmeth.4642 PMC6082636

[16] Choi, J., Gu, B., Chin, S. & Lee, J. S. Machine learning predictive model based on National data for fatal accidents of construction workers. *Autom. Constr.* **110**, 102974. 10.1016/J.AUTCON.2019.102974 (2020).

[17] Mohammed, J. & Mahmud, M. J. Selection of a machine learning algorithm for OSHA fatalities, 2020 IEEE Technology and Engineering Management Conference, TEMSCON (2020). (2020) 10.1109/TEMSCON47658.2020.9140142

[18] Assaad, R. & El-adaway, I. H. Determining Critical Combinations of Safety Fatality Causes Using Spectral Clustering and Computational Data Mining Algorithms, *J. Constr. Eng. Manag* **147** 04021035. 10.1061/(ASCE)CO.1943-7862.0002040. (2021).

[19] Baker, H., Hallowell, M. R. & Tixier, A. J. P. AI-based prediction of independent construction safety outcomes from universal attributes. *Autom. Constr.* **118**, 103146. 10.1016/J.AUTCON.2020.103146 (2020).

[20] Kang, K. & Ryu, H. Predicting types of occupational accidents at construction sites in Korea using random forest model. *Saf. Sci.* **120**, 226–236. 10.1016/J.SSCI.2019.06.034 (2019).

[21] Kale, Ö. A. & Baradan, S. Identifying factors that contribute to severity of construction injuries using logistic regression model. *Teknik Dergi*. **31**, 9919–9940. 10.18400/TEKDERG.470633 (2020).

[22] IIF Home : U.S. Bureau of Labor Statistics, (n.d.). (2024). https://www.bls.gov/iif/home.htm

[23] Zhu, J. et al. Developing predictive models of construction fatality characteristics using machine learning. *Saf. Sci.* **164**, 106149. 10.1016/J.SSCI.2023.106149 (2023).

[24] Chawla, N. V., Bowyer, K. W., Hall, L. O. & Kegelmeyer, W. P. Synthetic minority Over-sampling technique. *J. Artif. Intell. Res.* **16**, 321–357. 10.1613/JAIR.953 (2002).

[25] Seger, C. An investigation of categorical variable encoding techniques in machine learning: binary versus one-hot and feature hashing, Degree Project Technology (2018). https://urn.kb.se/resolve?urn=urn:nbn:se:kth:diva-237426 (accessed April 22, 2024).

[26] Koc, K., Ekmekcioğlu, Ö. & Gurgun, A. P. Integrating feature engineering, genetic algorithm and tree-based machine learning methods to predict the post-accident disability status of construction workers. *Autom. Constr.* **131**, 103896. 10.1016/J.AUTCON.2021.103896 (2021).

[27] Cerda, P., Varoquaux, G. & Kégl, B. Similarity encoding for learning with dirty categorical variables. *Mach. Learn.* **107**, 1477–1494. 10.1007/S10994-018-5724-2/TABLES/3 (2018).

[28] Le Chan, J. Y. et al. Mitigating the Multicollinearity Problem and Its Machine Learning Approach: A Review, *Mathematics ***10**, 1283 10. (2022). 10.3390/MATH10081283

[29] Aldhari, I. et al. Severity Prediction of Highway Crashes in Saudi Arabia Using Machine Learning Techniques, *Applied Sciences, ***13**, 233. (2023). 10.3390/APP13010233

[30] Pourroostaei Ardakani, S. et al. Road Car Accident Prediction Using a Machine-Learning-Enabled Data Analysis, *Sustainability,* **15**, 5939 15. (2023). 10.3390/SU15075939

[31] Ahmed, S., Hossain, M. A., Ray, S. K., Bhuiyan, M. M. I. & Sabuj, S. R. A study on road accident prediction and contributing factors using explainable machine learning models: analysis and performance. *Transp. Res. Interdiscip Perspect.* **19**, 100814. 10.1016/J.TRIP.2023.100814 (2023).

[32] Sufian, M. A., Varadarajan, J. & Niu, M. Enhancing prediction and analysis of UK road traffic accident severity using AI: integration of machine learning, econometric techniques, and time series forecasting in public health research. *Heliyon* **10**, e28547. 10.1016/J.HELIYON.2024.E28547 (2024). 38623197 10.1016/j.heliyon.2024.e28547 PMC11016589

[33] Han, M. et al. Reducing overfitting risk in Small-Sample learning with ANN: A case of predicting graduate admission probability. *Commun. Comput. Inform. Sci.* **2058 CCIS**, 404–419. 10.1007/978-981-97-1277-9_31 (2024).

[34] Uddin, S., Haque, I., Lu, H., Moni, M. A. & Gide, E. Comparative performance analysis of K-nearest neighbour (KNN) algorithm and its different variants for disease prediction. *Sci. Rep.* **12**, 1–11. 10.1038/S41598-022-10358- (2022). X;SUBJMETA=1041,639,692,699,705;KWRD=APPLIED+MATHEMATICS,DISEASES. 35428863 10.1038/s41598-022-10358-x PMC9012855

[35] Guo, G., Wang, H., Bell, D., Bi, Y. & Greer, K. KNN Model-Based approach in classification. *Lecture Notes Comput. Sci. (Including Subser. Lecture Notes Artif. Intell. Lecture Notes Bioinformatics)*. **2888**, 986–996. 10.1007/978-3-540-39964-3_62 (2003).

[36] Zhang, Z. Introduction to machine learning: k-nearest neighbors. *Ann. Transl Med.* **4**, 218–218. 10.21037/ATM.2016.03.37 (2016). 27386492 10.21037/atm.2016.03.37 PMC4916348

[37] Guido, R., Ferrisi, S., Lofaro, D. & Conforti, D. An Overview on the Advancements of Support Vector Machine Models in Healthcare Applications: A Review, Information, **15,** 235. (2024). 10.3390/INFO15040235

[38] Amaya-Tejera, N., Gamarra, M., Vélez, J. I. & Zurek, E. A distance-based kernel for classification via support vector machines. *Front. Artif. Intell.* **7**, 1287875. 10.3389/FRAI.2024.1287875/BIBTEX (2024). 38469159 10.3389/frai.2024.1287875 PMC10925654

[39] Abd, A. M. & Abd, S. M. Modelling the strength of lightweight foamed concrete using support vector machine (SVM), case studies in construction materials, **6,** 8–15. (2017). 10.1016/J.CSCM.2016.11.002

[40] Pereira, H. C., Bastos, A., Seco, A. & Antunes, F. Decision trees applied to injury severity in road accidents with one victim: focus on vulnerable users. *Proc. Institution Civil Eng. - Municipal Eng.* **177**, 38–49. 10.1680/JMUEN.23.00034 (2024).

[41] Kanyongo, W. & Ezugwu, A. E. Feature selection and importance of predictors of non-communicable diseases medication adherence from machine learning research perspectives. *Inf. Med. Unlocked*. **38**, 101232. 10.1016/J.IMU.2023.101232 (2023).

[42] Mistikoglu, G. et al. Decision tree analysis of construction fall accidents involving roofers. *Expert Syst. Appl.* **42**, 2256–2263. 10.1016/J.ESWA.2014.10.009 (2015).

[43] Warren, C. et al. Using a decision tree learning algorithm to classify injury status in adolescent males following return-to-activity criteria. *Gait Posture*. **81**, 383–384. 10.1016/J.GAITPOST.2020.08.091 (2020).

[44] Sun, Z. et al. An improved random forest based on the classification accuracy and correlation measurement of decision trees. *Expert Syst. Appl.* **237**, 121549. 10.1016/J.ESWA.2023.121549 (2024).

[45] Donick, D. & Lera, S. C. Uncovering feature interdependencies in high-noise environments with Stepwise Lookahead decision forests. *Sci. Rep.* **11**, 1–12. 10.1038/S41598-021-88571-3;SUBJMETA=1042,1046,117,639,705;KWRD=COMPUTATIONAL+SCIENCE,COMPUTER+SCIENCE,MATHEMATICS+AND+COMPUTING,SCIENTIFIC+DATA (2021). 33927260 PMC8085031

[46] Li, H., Lin, J., Lei, X. & Wei, T. Compressive strength prediction of basalt fiber reinforced concrete via random forest algorithm. *Mater. Today Commun.* **30**, 103117. 10.1016/J.MTCOMM.2021.103117 (2022).

[47] Sibindi, R., Mwangi, R. W. & Waititu, A. G. A boosting ensemble learning based hybrid light gradient boosting machine and extreme gradient boosting model for predicting house prices. *Eng. Rep.* **5**, e12599. 10.1002/ENG2.12599 (2023).

[48] Chakraborty, D., Elhegazy, H., Elzarka, H. & Gutierrez, L. A novel construction cost prediction model using hybrid natural and light gradient boosting. *Adv. Eng. Inform.* **46**, 101201. 10.1016/J.AEI.2020.101201 (2020).

[49] Lv, F. et al. An improved extreme gradient boosting approach to vehicle speed prediction for construction simulation of earthwork. *Autom. Constr.* **119**, 103351. 10.1016/J.AUTCON.2020.103351 (2020).

[50] Shehadeh, A., Alshboul, O., Al Mamlook, R. E. & Hamedat, O. Machine learning models for predicting the residual value of heavy construction equipment: an evaluation of modified decision tree, LightGBM, and XGBoost regression. *Autom. Constr.* **129**, 103827. 10.1016/J.AUTCON.2021.103827 (2021).

[51] Han, J., Shu, K. & Wang, Z. Predicting energy use in construction using extreme gradient boosting. *PeerJ Comput. Sci.* **9**, e1500. 10.7717/PEERJ-CS.1500/SUPP-1 (2023). 37705620 10.7717/peerj-cs.1500 PMC10496006

[52] Parsa, A. B., Movahedi, A., Taghipour, H. & Derrible, S. Kouros) Mohammadian, toward safer highways, application of XGBoost and SHAP for real-time accident detection and feature analysis. *Accid. Anal. Prev.* **136**, 105405. 10.1016/J.AAP.2019.105405 (2020). 31864931 10.1016/j.aap.2019.105405

[53] Lundberg, S. M. et al. From local explanations to global Understanding with explainable AI for trees. *Nat. Mach. Intell.* **2**, 56–67. 10.1038/S42256-019-0138-9 (2020). ;SUBJMETA=117,308,639,692,705,794;KWRD=COMPUTER+SCIENCE,MEDICAL+RESEARCH,SOFTWARE. 32607472 10.1038/s42256-019-0138-9 PMC7326367

[54] Štrumbelj, E. & Kononenko, I. Explaining prediction models and individual predictions with feature contributions. *Knowl. Inf. Syst.* **41**, 647–665. 10.1007/S10115-013-0679-X/METRICS (2014).

[55] Taiwo, R., Yussif, A. M., Ben Seghier, M. E. A. & Zayed, T. Explainable ensemble models for predicting wall thickness loss of water pipes. *Ain Shams Eng. J.* **15**, 102630. 10.1016/J.ASEJ.2024.102630 (2024).

[56] Taiwo, R. et al. An Integrated Approach of Simulation and Regression Analysis for Assessing Productivity in Modular Integrated Construction Projects, *Buildings,* **12**, 12 (2022). 10.3390/BUILDINGS12112018

